# Study on the tissue-specific distribution of triterpenoid saponins in *Panax japonicus* var. *major* based on metabolomics and transcriptomics

**DOI:** 10.1515/biol-2025-1300

**Published:** 2026-04-08

**Authors:** Ziyue Wang, Yue Xu, Yuze Li, Dongdong Zhang, Xiaomei Song, Wenli Huang

**Affiliations:** College of Pharmacy, Shaanxi University of Chinese Medicine, Xianyang, 712046, China; Key Laboratory of “Taibaiqiyao” Research and Applications, Shaanxi University of Chinese Medicine, Xianyang, 712046, China

**Keywords:** metabolome, transcriptome, *P. japonicus* var. *major*, tissue-specific saponin distribution

## Abstract

*Panax japonicus* C. A. Mey. var. *major* (Burk.) C. Y. Wu et K. M. Feng is an essential medicinal plant in the genus *Panax* of the family Araliaceae. Research on the triterpenoid saponin biosynthesis pathway in *P. japonicus* var. *major* is currently limited. This study employed widely targeted metabolomics technology based on UPLC-MS/MS to detect 384 metabolites in the rhizomes, internodes, leaves, petioles, and stems of *P. japonicus* var. *major*. Triterpenoid saponins exhibited tissue-specific accumulation: the underground parts mostly contained oleanane-type saponins. In contrast, the aboveground parts mostly contained dammarane-type saponins. Transcriptome sequencing of rhizomes and leaves in *P. japonicus* var. *major* identified 26,988 differentially expressed genes and 87 key enzyme genes involved in triterpenoid saponin biosynthesis. β-AS was remarkably upregulated in rhizomes, confirming the specific accumulation of oleanane-type triterpenoid saponins in underground rhizomes, which was corroborated by metabolomic data. This study reveals the tissue-specific regulatory characteristics of oleanane-type triterpenoid saponin biosynthesis, laying a theoretical foundation for further research on related metabolic pathways.

## 1 Introduction

As one of the distinctive traditional Chinese herbal resources, Taibai Qi Yao, *Panax japonicus* C. A. Mey. var. *major* (Burk.) C. Y. Wu et K. M. Feng (*P. japonicus* var. *major*) is an essential medicinal plant in the genus *Panax* of the family Araliaceae. *P. japonicus* var. *major* contains saponins, polysaccharides, phenolic acids, volatile oils, amino acids, and other active substances, as well as a variety of trace elements [1]. Triterpenoid saponins are one of the principal medicinal components of this herb and exert remarkable pharmacological activities, such as anti-tumor, anti-inflammatory, and hepatoprotective effects, alongside regulatory functions in the nervous, immune, and hematopoietic systems [2]. The rhizomes of *P. japonicus* var. *major* are its conventional medicinal part, while its leaves are also widely applied in folk medicine. The leaves possess the effects of relieving cough, moistening the throat, alleviating summer-heat, and tonifying the body, and can also be brewed as herbal tea for drinking [3].

Medicinal plants of the genus *Panax* contain saponins not only in underground roots or rhizomes but also in the aboveground parts, such as stems and leaves. Similarly, saponin content varies significantly among different plant species in aboveground parts. The *Panax Ginseng* leaves and stems contain saponins similar to those in the roots and rhizomes, primarily dammarane-type saponins [4]. Both the roots and leaves of *Panax notoginseng* are rich in dammarane-type saponins. Still, the roots also contain protopanaxadiol (PPD)-type and protopanaxatriol (PPT)-type saponins, while the leaves contain PPD-type saponins, showing significant compositional differences [5]. The primary saponins present in American ginseng are dammarane-type saponins [6]. *P. japonicus* var. *major* exhibits a diverse array of triterpenoid saponins. Its rhizomes display a unique co-enrichment pattern of both oleanane-type and dammarane-type saponins [5]. This chemical characteristic provides a more complete and systematic approach to exploring triterpenoid saponin biosynthesis pathways. It also facilitates research into the regulatory mechanisms governing the biosynthesis of different triterpenoid saponin types. Therefore, *P. japonicus* var. *major* serves as a unique material for studying triterpenoid saponin biosynthesis and regulation.

**Figure 1:**
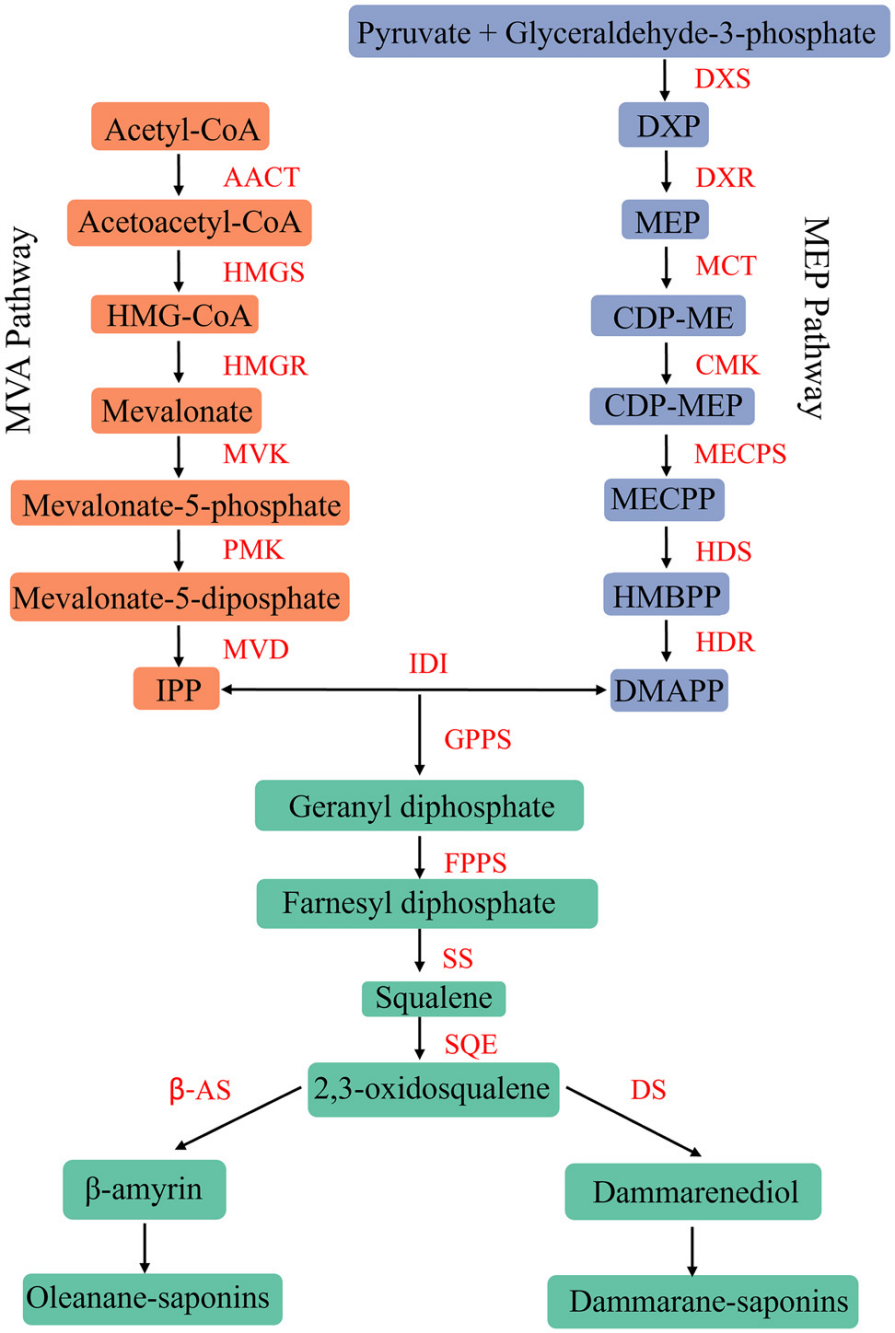
The triterpenoid saponin biosynthetic pathway. HMG-CoA, 3-hydroxy-3-methylglutatryl-CoA; IPP, isopentenyl diphosphate; AACT, acetyl-CoA-acetyltransferase; HMGS, 3-hydroxy-3-methylglutatryl-CoA synthase; HMGR, 3-hydroxy-3-methylglutatryl-CoA reductase; MVK, mevalonate kinase; PMK, phosphomevalonate kinase; MVD, mevalonate-5-diphosphate decarboxylase; DXP, 1-deoxy-d-xylulose-5-phosphate; MEP, 2-c-methyl-d-erythritol 4-phosphate; CDP-ME, 4-(cytidine 5′-diphospho)-2-c-methyl-d-erythritol; CDP-MEP, 2-phospho-4-(cytidine 5′-diphospho)-2-c-methyl-d-erythritol; MECPP, 2-c-methyl-d-erythritol 2,4-cyclodiphosphate; HMBPP, 4-hydroxy-3-methylbut-2-enyl-diphosphate; DMAPP, dimethylallyl diphosphate; DXS, 1-deoxy-d-xylulose-5-phosphate synthase; DXR, 1-deoxy-d-xylulose-5-phosphate reductoisomerase; MCT, 2-c-methyl-d-erythritol 4-phosphate cytidylyltransferase; CMK, 4-diphosphoeytidyl-2-c-methyl-d-erythritol kinase; MECPS, 2-c-methyl-d-erythritol 2,4-cyclodiphosphate synthase; HDS, 4-hydroxy-3-methylbut-2-enyl-diphosphate synthase; HDR, 4-hydroxy-3-methylbut-2-enyl diphosphate reductase; IDI, isopentenyl-diphosphate delta-isomerase; GPPS, geranyl diphosphate synthase; FPPS, farnesyl diphosphate synthase; SS, squalene synthase; SQE, squalene monoxgenase; β-AS, β-amyrin synthase; DS, dammaranediol synthase.

There are two main pathways for triterpenoid saponins biosynthesis: one is the mevalonate (MVA) pathway, which is present in the cytoplasm and mitochondria; the other, the 2-c-methyl-d-erythritol-4-phosphate (MEP) pathway, is present in the plastids [7]. The triterpenoid saponin biosynthesis pathway can be divided into three parts. (1) Biosynthesis of the triterpenoid saponin precursor substances isopentenyl diphosphate (IPP) and dimethylallyl diphosphate (DMAPP), formation of IPP and DMAPP by the MVA and MEP pathways, 3-hydroxy-3-methylglutaryl-CoA reductase (HMGR) in the MVA pathway and 1-deoxy-d-xylulose 5-phosphate synthase(DXS)in the MEP pathway, 1-deoxy-d-xylulose 5-phosphate reductoisomerase (DXR) are key enzymes for precursor biosynthesis. (2) The IPP and DMAPP in the presence of the enzymes geranyl diphosphate synthase (GPPS), farnesyl diphosphate synthase (FPPS), squalene synthase (SS), and squalene monoxgenase (SQE) to generate 2,3-oxidosqualene, which is a common prerequisite for the production of triterpenoid saponins and phytosterols [8]. DMAPP and IPP react under the catalysis of GPPS to form the monoterpene precursor geranyl diphosphate (GPP). Furthermore, under the catalysis of FPPS, IPP and GPP form farnesyl diphosphate (FPP), which serves as the backbone of sesquiterpenes. Geranylgeranyl diphosphate synthase (GGPPS) catalyses the combination of three IPPs with one DMAPP to form geranylgeranyl diphosphate (GGPP), a precursor for diterpenes. FPP and GGPP can further polymerise to form triterpenes and tetraterpenes [9]. (3) The biosynthesis of different types of triterpenoid saponins. β-amyrin synthase (β-AS) catalyzes the formation of β-amyrin (oleanolic acid precursor) from 2,3-oxidosqualene, followed by the biosynthesis of oleanane-type triterpenoid saponins under the action of glycosyltransferases; dammarenediol synthase (DS) catalyzes the cyclization of 2,3-oxidosqualene to dammaranediol, and DS is a key enzyme for dammarane-type triterpenoid saponins [10] (Figure 1).

At present, many studies focus on the biosynthesis of triterpenoid saponins in medicinal plants of the genus *Panax*, such as *Panax ginseng* and *P. notoginseng*, as well as the accumulation of saponins in plants. Liu et al. [11] screened out the *NAC* gene *PgNAC41-2*, which is closely associated with ginsenoside biosynthesis. In the positive hairy roots overexpressing *PgNAC41-2*, the contents of Re, Rc, Rb2, PPT, Rh2, and total saponins were significantly higher than those in the wild type, demonstrating that *PgNAC41-2* acts as a positive regulator of ginsenoside biosynthesis. Liu et al. [12] identified the transcription factor *PgMYB2* in the adventitious roots of *P. ginseng*, which showed the highest expression level under methyl jasmonate (MeJA) induction. The binding site between *PgMYB2* and its target gene *DS* was verified via electrophoretic mobility shift assay (EMSA). Transient overexpression assays in plants showed that *PgMYB2* may positively regulate *PgDDS* transcription. Jiang et al. [13] found that the *PgCYP309* gene responded to MeJA induction and participated in ginsenoside biosynthesis, as evidenced by its induction in *P. ginseng* adventitious roots. Analysis of ginsenoside contents and relative gene expression levels in positive asexual lines of ginseng hairy roots showed that the contents of PPD-type, PPT-type ginsenosides, and the PPT-type monomeric ginsenosides Re and Rg2, as well as markedly upregulated expression of *PgCYP309* and *PgCYP716A53v2*. The *PgCYP309* gene promotes ginsenoside biosynthesis, providing preliminary evidence for its role in the biosynthesis of dammarane-type ginsenosides. Man et al. [14] reported that suppressing the expression of *PnMYB4* in *P. notoginseng* calli significantly increased saponin content and the transcript levels of saponin biosynthetic genes, including *PnSS*, *PnSE*, and *PnDS*. *PnMYB4* and the transcriptional activator *PnMYB1* can interact with *PnbHLH*, a positive regulator of saponin biosynthesis, to jointly modulate the biosynthetic process. Lei et al. [15] demonstrated that overexpression of the transcription factor *PnMYB1* in *P. notoginseng* cells significantly enhanced the expression of *PnDS* and *PnSE*, thereby increasing the contents of ginsenosides R1, Rg1, Re, Rb1, and Rd. Hou et al. [16] functionally characterized six glycosyltransferases (UGTs) from *P. notoginseng*, including two novel enzymes: *PnUGT71A3* and *PnUGT94M1*. *PnUGT71A3* catalyzes the C6-hydroxyl glycosylation of PPT and F1 to produce Rh1 and Rg1, respectively, as well as the C20-hydroxyl glycosylation of the PPD-type ginsenoside Rg3 to generate Rd. Particularly, *PnUGT94M1* is a UDP-*β*-l-rhamnose (UDP-Rha)-dependent enzyme that regioselectively rhamnosylates the C2′-hydroxyl group of the C6-linked glucose moiety in PPT-type ginsenosides Rg1 and Rh1, yielding Re and Rg2, respectively.

As a traditional Chinese medicinal herb, studies on *P. japonicus* var. *major* have mainly focused on the isolation of chemical constituents, the evaluation of pharmacological activities, and germplasm resource research. However, investigations into the biosynthesis of triterpenoid saponin in this species remain relatively scarce. Several key genes involved in the triterpenoid saponin biosynthetic pathway, including *HMGR*, *β-AS*, and *FPS*, have been cloned and analyzed for their expression patterns [17], [18], [19]. Previous studies have verified that the key enzymes PjFPS and PjβAS positively regulate the biosynthesis of total saponins in *P. japonicus* var. *major*. In cell lines of this herb overexpressing both *PjFPS* and *PjβAS*, the contents of ginsenosides Rb1 and Rd, together with chikusetsusaponin IVa and chikusetsusaponin IV, were increased [20]. Regarding key genes associated with post-modification, Zhang et al. [21] identified at least four UGT genes potentially involved in oleanane-type triterpenoid saponin biosynthesis in the rhizomes and internodes of *P. japonicus* var. *major*.

Compared with other medicinal herbs of the genus *Panax*, such as *P. ginseng* and *P. notoginseng*, studies on the secondary metabolic pathways of *P. japonicus* var. *major* remain relatively scarce. Even less is understood regarding the biosynthesis and regulatory mechanisms underlying the accumulation of different triterpenoid saponins in its aboveground and underground parts. To clarify the comprehensive differences and distribution of triterpenoid saponins in various tissues of *P. japonicus* var. *major*, reveal the association between ginsenoside biosynthesis and metabolites, and screen differentially expressed genes (DEGs) between its rhizomes and leaves, this study performed metabolomic and transcriptomic analyses of different tissues of this herb. The results will provide new insights into the regulatory mechanisms governing the biosynthesis of different triterpenoid saponin types in the aboveground and underground parts of *P. japonicus* var. *major*.

## 2 Materials and methods

### 2.1 Plant materials

The plants of *P. japonicus* var. *major* were collected from the Red River Resorts, Meixian County, Baoji City, Shaanxi Province. The plants were separated into rhizomes (ZA), internodes (ZB), leaves (ZC), petioles (ZD), and stems (ZE). The treated tissues were immediately frozen in liquid nitrogen and then stored at −80 °C until analysis. Three biological replicates were prepared for each tissue in this study.

### 2.2 Metabolomics analysis

The rhizomes (ZA), internodes (ZB), leaves (ZC), petioles (ZD), and stems (ZE) of *P. japonicus* var. *major* were vacuum freeze-dried in a lyophilizer (Scientz-100F, China), and the lyophilized samples were ground using a grinder (Retsch-MM 400, Germany) at 30 Hz for 1.5 min 100 mg of lyophilized powder was weighed and dissolved in 0.6 mL of 70 % methanol extract, and the extracts were refrigerated at 4 °C overnight. The extract was vortexed six times to increase the extraction rate. The extract was placed in a freezing centrifuge (Sigma, Germany) and centrifuged at 10,000 g for 10 min. The supernatant was filtered through a 0.22 μm microporous membrane, and the filtrate was saved for UPLC-MS/MS analysis.

Sample data were collected using UPLC-MS/MS, mass spectral data were corrected for mass peaks, and secondary spectral information was used for substance characterization. Quantification of metabolites was performed using multiple reaction monitoring (MRM) analysis with a triple quadrupole mass spectrometer. Samples were subjected to sample quality control analysis, principal component analysis (PCA), cluster analysis, orthogonal partial least squares discriminant analysis (OPLS-DA). The predictive parameters used to evaluate the OPLS-DA model are R2Y and Q2. R2Y represents the model’s explanatory power for the Y matrix, while Q2 indicates the model’s predictive capability. The closer these two metrics are to 1, the more stable and reliable the model is. A model with Q2 > 0.5 is considered valid, and one with Q2 > 0.9 is considered excellent. To identify differential metabolites, the variable importance in the projection (VIP) from the OPLS-DA model was used as the screening criterion. Metabolites with fold change ≥2 and fold change ≤0.5, and VIP ≥1 were selected as differential metabolites. The PCA, OPLS-DA, heatmaps, bubble plots, and circle plots were generated using Metware Cloud, a free online data analysis platform (https://cloud.metware.cn).

### 2.3 Transcriptomics analysis

Based on metabolomic results, representative leaves (ZC) from the aboveground parts with the most significant differences and representative rhizomes (ZA) from underground parts were selected for transcriptome sequencing. The material sources were the same as those of the rhizomes (ZA) and leaves (ZC) described in Section 2.1.

#### 2.3.1 RNA library construction and sequencing

To ensure RNA quality, the samples were tested using the following methods. Only after passing the tests can the construction of the library proceed. To obtain high-quality RNA, the samples were tested using the following methods. RNA concentration was measured using a Qubit 2.0 Fluorometer, and RNA integrity was evaluated using an Agilent 2100 Bioanalyzer. After the RNA samples passed the tests, library construction and transcriptome sequencing were carried out. After the library construction was completed, its quality needs to be tested. Only when the test results meet the requirements can the sequencing be carried out on the machine. Qubit 2.0 was used for accurate quantification of the library’s effective concentration (>2 nM); Agilent 2100 was used to evaluate the integrity of the library. After the library passes the test, libraries are pooled based on the target data volume to be downloaded from the machine, and sequencing was performed on the Illumina HiSeq platform.

#### 2.3.2 Bioinformatics analysis

The raw data were processed to obtain high-quality sequencing reads, which were spliced to generate the transcriptome, and the high-quality reads were then compared with the transcriptome to calculate gene expression. Then used BLAST software to compare the Unigene sequence with NR, Swiss-Prot, KOG, Trembl, KEGG, and GO databases; used HMMER software to compare the Unigene amino acid sequence with the Pfam database to obtain Unigene annotation information; and then screened the DEGs. The annotation results based on the NR, KOG, KEGG, and GO databases were generated using Metware Cloud, a free online data analysis platform (https://cloud.metware.cn).

#### 2.3.3 Screening of differentially expressed genes

DEGs were identified by comparing RNA-Seq data from the two tissues: rhizomes (ZA) and leaves (ZC). The DESeq2 R package is suitable for intergroup differential expression analysis of samples with biological replicates. DESeq2 has two key settings: (1) it requires unnormalized read count data; (2) The Benjamini-Hochberg method is employed to perform multiple hypothesis testing correction on the significance probability (*P*-value), yielding the false discovery rate (FDR). DEGs were screened using the thresholds of |log2FoldChange|≥1 and FDR < 0.05.

## 3 Results

### 3.1 Metabolomics analysis

#### 3.1.1 Sample quality control analysis

**Figure 2:**
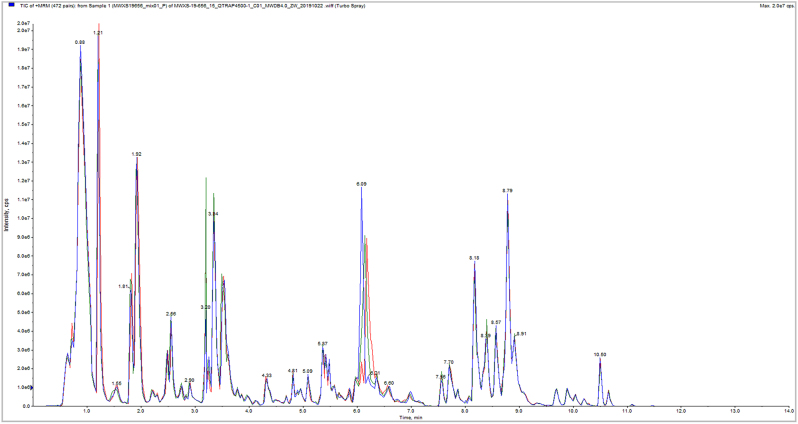
TIC overlap detection by QC samples essence spectrum (positive ion mode).

**Figure 3:**
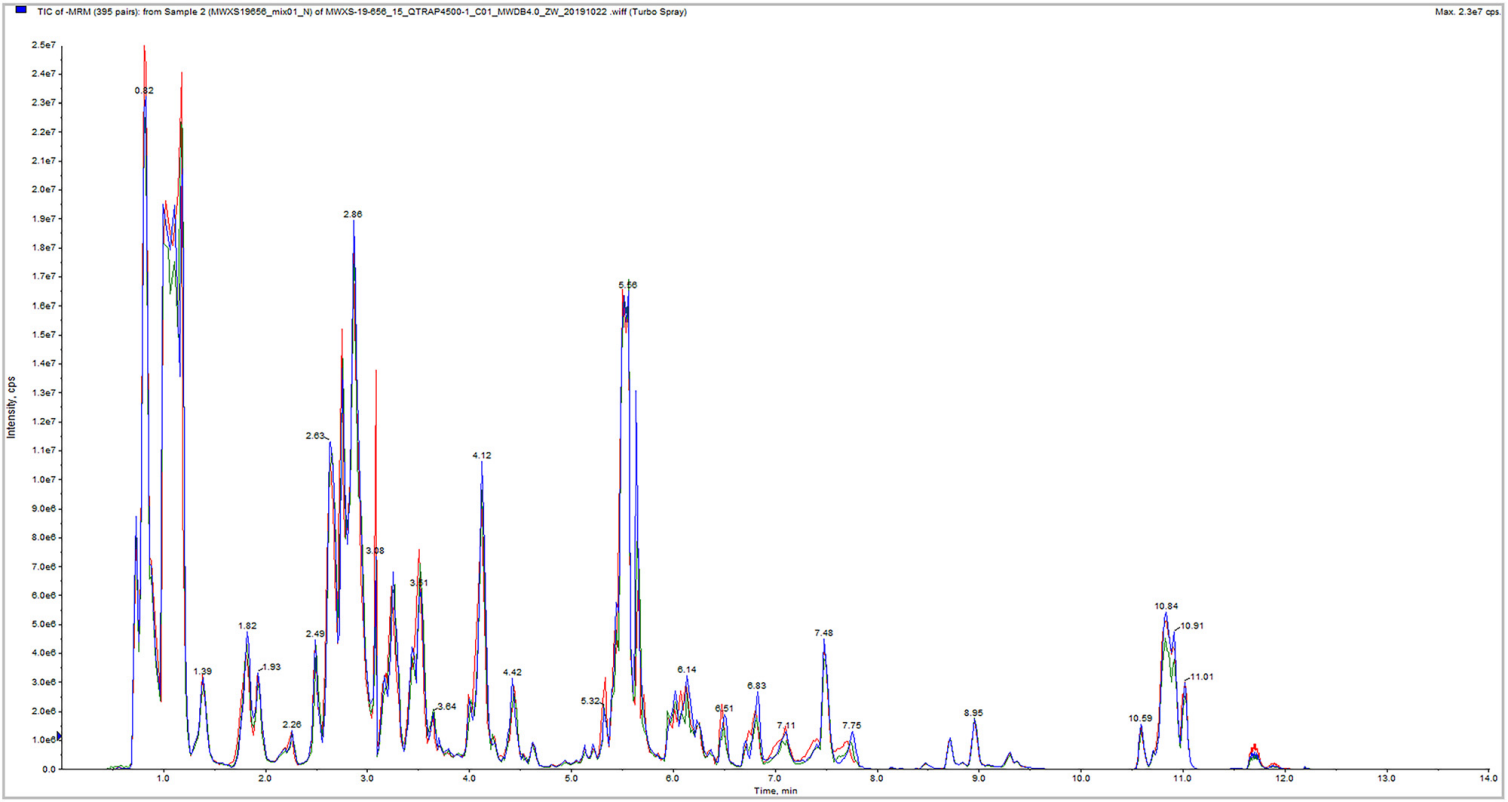
Overlay of TIC for mass spectrometry detection of QC samples (negative ion mode).

Total ion current (TIC) plots show high overlap of metabolite detection curves, with well-aligned retention times and peak intensities. This confirms excellent instrument stability and ensures the reliability and reproducibility of the acquired data, supporting the use of the resulting metabolomic data for subsequent analyses (Figures 2 and 3).

#### 3.1.2 Principal component analysis

**Figure 4:**
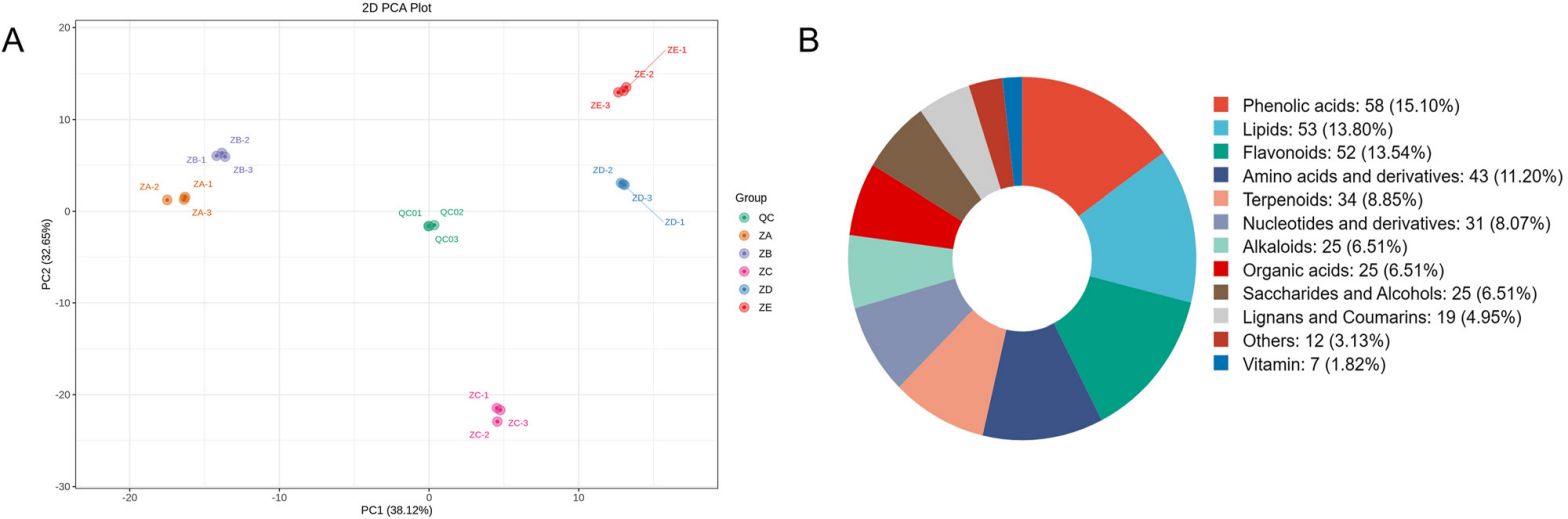
Distribution and analysis of metabolites in different tissues of *P.* *japonicus* var. *major*. (A) PCA analysis of metabolome profiles of different tissues. Each point represents a sample, with colours indicating tissue types: QC (green, quality control), ZA (orange, rhizomes), ZB (purple, internodes), ZC (pink, leaves), ZD (blue, petioles), and ZE (red, stems) (B) type and quantity of metabolites.

PCA verified the reliability of the sample data processing. The results revealed significant metabolic differences among different tissues of *P. japonicus* var. *major*, with distinct differentiation between aboveground and underground parts (Figure 4A).

#### 3.1.3 Metabolite identification notes

A total of 384 metabolites were detected and identified based on the matrix of retention time, mass-to-charge ratio, and peak intensity. The metabolites detected in different tissue samples were classified and counted. Among the metabolites obtained, phenolic acids, lipids, and flavonoids were the most abundant, followed by amino acids and their derivatives, terpenoids, and nucleotides and their derivatives (Figure 4B).

**Figure 5:**
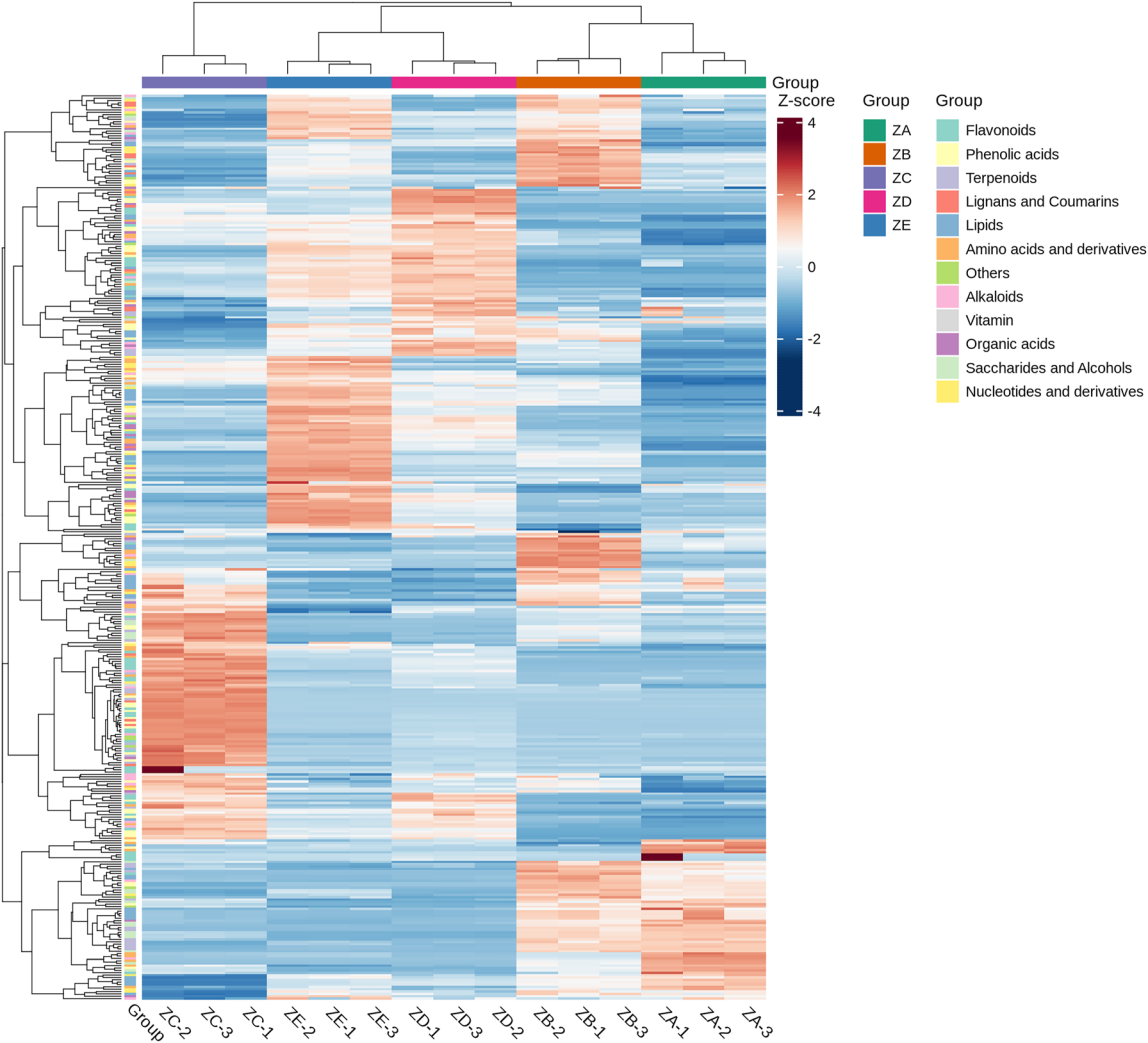
Hierarchical clustering heatmap of differential metabolites across tissues of *P. japonicus* var. *major*. The heatmap displays the relative abundance of differential metabolites (rows) in different tissue samples (columns). The colour scale represents the Z-score normalised abundance, with red indicating high abundance and blue indicating low abundance. Tissue types are labelled at the top: ZA (rhizomes), ZB (internodes), ZC (leaves), ZD (petioles), and ZE (stems). The right-side bar shows the metabolite classes, revealing distinct accumulation patterns across tissues.

Perform cluster analysis on metabolites from different tissues of *P. japonicus* var. *major* (Figure 5). From the figure, it can be seen that the relative content of metabolites varied significantly in different tissues. The main types of compounds contained in the rhizomes (ZA) were flavonoids, lipids, terpenoids, saccharides and alcohols, amino acids and derivatives, phenolic acids, and nucleotides and derivatives; the main types of compounds in the internodes (ZB) were lignans and coumarins, nucleotides and derivatives, phenolic acids, terpenoids, lipids, organic acids, alkaloids, and saccharides and alcohols; the main types of compounds in the leaves (ZC) were flavonoids, lipids, phenolic acids, terpenoids, amino acids and derivatives, lignans and coumarins, saccharides and alcohols; the main types of compounds in the petioles (ZD) were flavonoids, lipids, phenolic acids, terpenoids, amino acids and their derivatives, organic acids, lignans and coumarins; the main types of compounds in the stems (ZE) were types are lipids, organic acids, nucleotides and derivatives, amino acids and derivatives, phenolic acids, flavonoids, lignans and coumarins.

#### 3.1.4 OPLS-DA analysis

**Figure 6:**
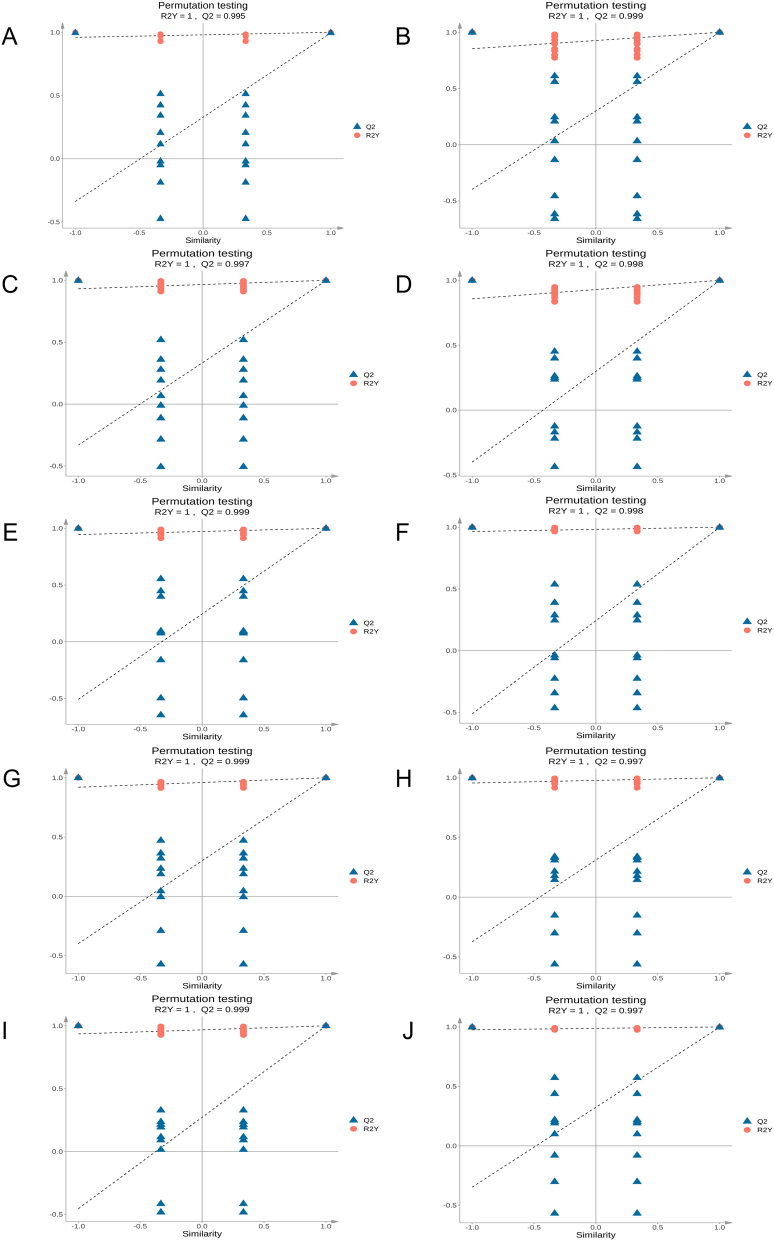
The OPLS-DA permutation test plot for inter-group comparison. The *x*-axis represents the displacement retention, and the *y*-axis represents the value of R2Y or Q2. (A–J) are the permutation test plots of OPLS-DA of ZA vs ZB, ZA vs ZC, ZA vs ZD, ZA vs ZE, ZB vs ZC, ZB vs ZE, ZC vs ZD, ZC vs ZE, ZD vs ZE.

Multidimensional statistics were used to establish reliable mathematical models to summarize and analyze the metabolite data of the study samples. Different tissue parts were divided into 10 groups for comparative analysis, and OPLS-DA mathematical models were established and analyzed. The results showed that the R2Y and Q2 values of the OPLS-DA models for the ten-group comparison were high, indicating excellent predictive ability and reliability, and that the trends in metabolite changes among the groups were well represented. The OPLS-DA models were validated using 200 permutations. The results showed that the R2Y and Q2 values of the validated models did not exceed those of the original models, indicating that the models did not exhibit overfitting and that different metabolites could be screened based on VIP value analysis (Figure 6).

#### 3.1.5 Screening and characterization of differential metabolites

Differential metabolites were identified through pairwise comparisons of rhizomes (ZA), internodes (ZB), leaves (ZC), petioles (ZD), and stems (ZE), based on fold change ≥2 or fold change ≤0.5 and VIP ≥1. Results revealed the highest number of differential metabolites in the internodes (ZB) and leaves (ZC) comparison group, predominantly enriched in the internodes (ZB) (Table 1).

**Table 1:** Statistical table on the number of differential metabolites.

Group	All sig diff	Down	Up	Group	All sig diff	Down	Up
ZA_vs_ZB	144	35	109	ZB_vs_ZD	229	105	124
ZA_vs_ZC	243	92	151	ZB_vs_ZE	202	85	117
ZA_vs_ZD	243	78	165	ZC_vs_ZD	191	84	107
ZA_vs_ZE	249	75	174	ZC_vs_ZE	238	109	129
ZB_vs_ZC	251	126	125	ZD_vs_ZE	119	68	51

#### 3.1.6 KEGG pathway analysis

**Figure 7:**
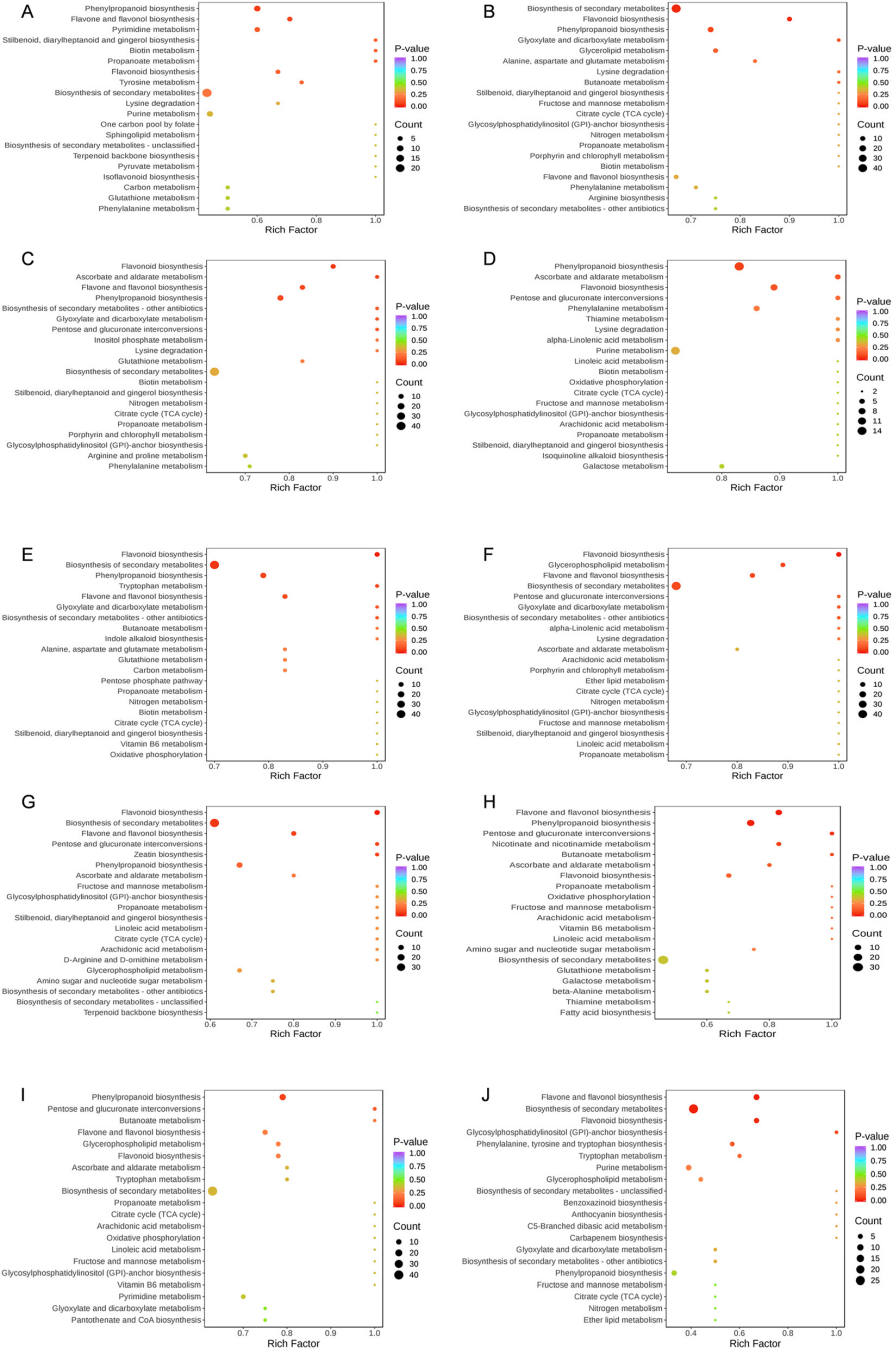
KEGG enrichment bubble plot of 10 groups of differential metabolites. The *x*-axis represents the rich factor, and the *y*-axis represents the pathway name; the colour of the dots indicates the magnitude of the *P*-value; the size of the dots indicates the number of differential metabolites enriched; (A–J) show the enrichment of differentially metabolites in the top 20 metabolic pathways between ZA vs ZB, ZA vs ZC, ZA vs ZD, ZA vs ZE, ZB vs ZC, ZB vs ZD, ZB vs ZE, ZC vs ZD, ZC vs ZE, ZD vs ZE.

The KEGG database was utilized to annotate the differential metabolites in the 10 comparison groups (Figure 7). The differential metabolites were mainly enriched in Biosynthesis of secondary metabolites, Flavonoid biosynthesis, Flavone and flavonol synthesis, Phenylpropanoid biosynthesis, and Pentose and glucuronate interconversion.

#### 3.1.7 Tissue accumulation of upstream carbon metabolism for triterpenoid saponin biosynthesis

**Figure 8:**
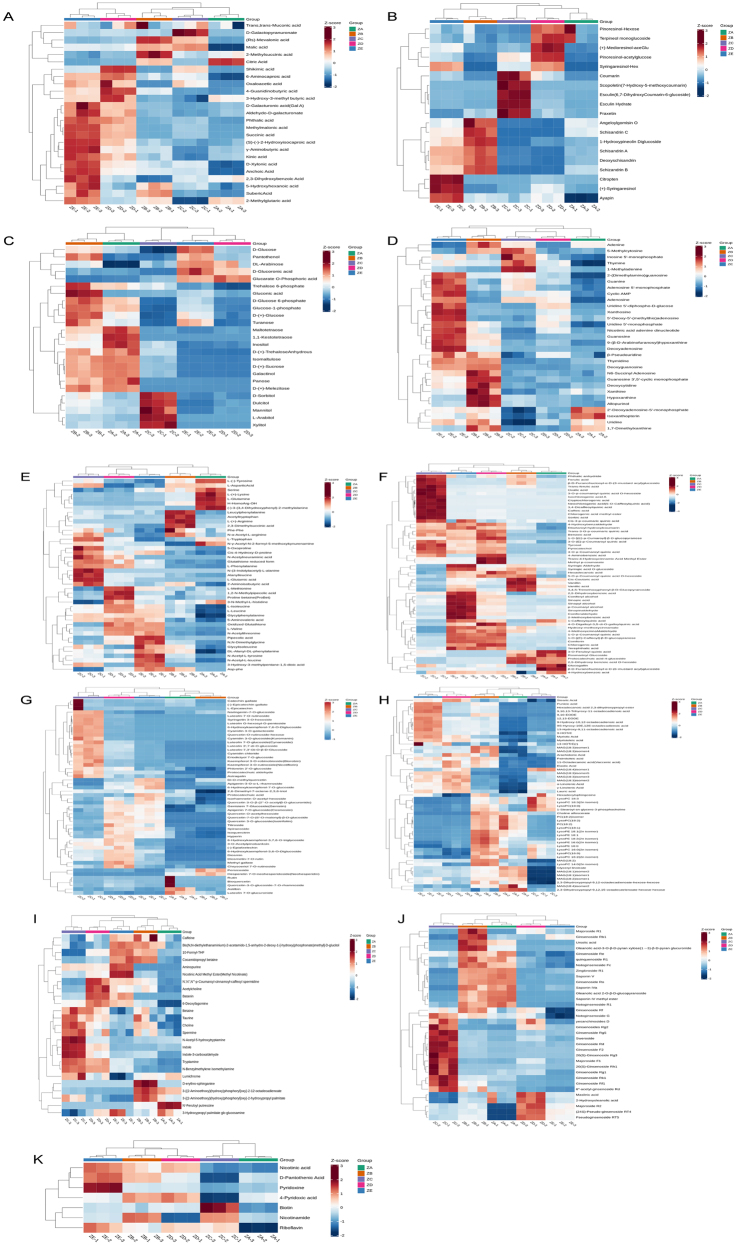
Heatmap of clustering for various compound types. (A–K) represent organic acids, lignans and coumarins, sugars and alcohols, nucleotides and derivatives, amino acids and derivatives, phenolic acids, flavonoids, lipids, alkaloids, terpenoids, and vitamin compounds, respectively.

In this study, metabolites related to carbon metabolism were screened and analysed, among which those related to glucose metabolism and glycolysis were: d-(+)-sucrose and d-glucose-6-phosphate accumulated in the rhizomes (ZA) and internodes (ZB), while D-(+)-glucose mainly accumulated in the internodes (ZB) and the stems (ZE). In the tricarboxylic acid (TCA) cycle, succinic acid accumulated in the leaves (ZC), citric acid accumulated in the rhizomes (ZA) and internodes (ZB). The shikimic acid-phenylpropanoid metabolism also falls within the scope of carbon metabolism. The accumulation of phenylpropanoids in the five tissues. These compounds include coniferaldehyde, sinapyl alcohol, coniferyl alcohol, *p*-coumaryl alcohol, sinapic acid, sinapinaldehyde, coumarin, caffeic acid, and l-phenylalanine, which are mainly concentrated in the petioles (ZD) and the stems (ZE); coumarin and caffeic acid are mainly concentrated in the leaves (ZC), while l-phenylalanine accumulated in the leaves (ZC) and petioles (ZD); ferulic acid accumulated in the internodes (ZB) and the leaves (ZC). Regarding biosynthetic accumulation of flavonoids, only rhizomes (ZA) and internodes (ZB) showed similar accumulation patterns, whereas distinct profiles were observed in the other tissues. These compounds include: (−)-epiafzelechin, naringenin-7-o-glucoside, l-epicatechin, and luteolin, mainly accumulated in the leaves (ZC); apigenin 7-o-glucoside and quercetin in the petioles (ZD). Regarding the metabolites of amino acid biosynthesis, accumulation varied across the five tissues: l-(−)-tyrosine and l-glutamine mainly accumulated in the rhizomes (ZA) and internodes (ZB), whereas shikimic acid, l-tryptophan, indole, l-glutamic acid, and l-phenylalanine mainly accumulated in the aboveground parts. The tissue-specific accumulation patterns of metabolites associated with sugar metabolism, glycolysis, and the TCA cycle indicate significant variations in primary carbon metabolism across tissues. These primary metabolic pathways provide essential energy for triterpenoid saponin biosynthesis, thereby indirectly influencing the synthesis and accumulation of tissue-specific terpenoid products. To better observe these patterns, cluster heat maps were generated based on metabolite compound types. (Figure 8).

#### 3.1.8 The accumulation of triterpenoid saponins in *P. japonicus* var. *major* in different tissues

The research found that the two main types of saponins, oleanane-type and dammarane-type of *P. japonicus* var. *major* have tissue-specific distribution phenomena. To further study this situation, 34 terpenoids were individually analysed by cluster analysis (Figure 8J). The oleanane-type triterpenoid saponins are concentrated in the underground parts (rhizomes (ZA) and internodes (ZB)), while the dammarane-type triterpenoid saponins are present in large quantities in the leaves (ZC) and a small amount in petioles (ZD) and stems (ZE).

A two-by-two comparison of five tissue parts showed that in the underground parts, rhizomes (ZA) and internodes (ZB) were present. Among these, including ginsenoside Rb1, 6″-acetyl-ginsenoside Rd, ginsenoside Rf1, majoroside R1, majoroside R2, (24S)-pseudo-ginsenoside RT4, ginsenoside Rg5, 20(S)-ginsenoside Rg3, ginsenoside Rg2, pseudoginsenoside RT5, ginsenoside F2, oleanolic acid-3-o-β-d-pyran xylose (1 → 3)-β-d-pyran glucuronide, which are 12 triterpenoid saponins were significantly different (Supplementary Table 1). Saponins were mainly accumulated in internodes (ZB).

Comparing the rhizomes (ZA), leaves (ZC), petioles (ZD) and stems (ZE), the numbers of triterpenoid saponins with differences were 26, 24 and 25 (Supplementary Tables 2–4), respectively, indicating that the triterpenoid saponins had significant differences aboveground and underground, and it was clearly seen that the oleanane-type triterpenoid saponins were concentrated in the rhizomes (ZA); the dammarane-type saponins were mainly accumulated in leaves (ZC); however, a few PPT-type triterpenoid saponins were present in the rhizomes (ZA), including notoginsenoside R1 and ginsenoside Re; furthermore, comparing the rhizomes (ZA) with the petioles (ZD) and stems (ZE), we found that the most of the dammarane-type saponin were mainly accumulated in the rhizomes (ZA). Similarly, comparing internodes (ZB) to leaves (ZC), petioles (ZD), and stems (ZE), the numbers of differential saponins were 23, 21, and 22, respectively (Supplementary Tables 5–7). Comparisons between internodes (ZB) and leaves (ZC) showed oleanane-type saponins were mainly accumulated in internodes (ZB), while dammarane-type saponins were mainly accumulated in leaves (ZC). Comparisons between internodes (ZB) and petioles (ZD) revealed that both oleanane-type and dammarane-type saponins were mainly accumulated in petioles (ZD). Distribution patterns of the two saponin types in comparisons between internodes (ZB) and stems (ZE) were consistent with those between internodes (ZB) and petioles (ZD).

Finally, the aboveground parts of the leaves (ZC), petioles (ZD), and stems (ZE) were compared to identify differential characteristics among the tissues. There were 20 differential saponins between the leaves (ZC) and petioles (ZD) (Supplementary Table 8). The differential saponins of leaves (ZC) and petioles (ZD) include five types of oleanane-type saponins: ginsenoside Ro, zingbroside R1, chikusetsusaponin IVa, chikusetsusaponin V, and oleanolic acid 2-o-β-d-pyran glucopyranoside. These five saponins were mainly accumulated in petioles (ZD). Dammarane-type triterpenoid saponins such as ginsenoside Rg1, 6″-acetyl-ginsenoside Rd, and ginsenoside Rd are primarily concentrated in the leaves (ZC). There were 25 differential saponins between the leaves (ZC) and stems (ZE) (Supplementary Table 9). Oleanane-type saponins were more concentrated in the stems (ZE), while dammarane-type saponins were more concentrated in the leaves (ZC). Finally, 15 differential saponins were found between the petioles (ZD) and stems (ZE) (Supplementary Table 10). Three oleanane-type saponins, namely zingibroside R1, oleanolic acid-3-o-β-d-pyran xylose (1 → 3)-β-d-pyran glucuronide, and oleanolic acid 2-o-β-d-pyran glucopyranoside, were concentrated in the stems (ZE); whereas dammarane-type saponins, except ginsenoside Rb1, were more concentrated in the petioles (ZD).

The results showed that the triterpenoid saponin components were distributed differently across the different tissue parts of *P. japonicus* var. *major*. Among these, most of the oleanane-type saponins, such as chikusetsusaponin IVa, ginsenoside Ro, zingbroside R1, oleanolic acid-3-o-β-d-pyran xylose (1 → 3)-β-d-pyran glucuronide, and oleanolic acid 2-o-β-d-pyran glucopyranoside in *P. japonicus* var. *major* are distributed with higher concentrations in the underground parts (rhizomes (ZA) and internodes (ZB)) than in the aboveground parts (leaves (ZC), petioles (ZD), and stems (ZE)). Most dammarane-type triterpenoid saponins were mainly accumulated in leaves (ZC), at lower concentrations in petioles (ZD) and stems (ZE). A few PPT-type saponins, including notoginsenoside R1 and ginsenoside Re, were mainly accumulated in the rhizomes (ZA). According to metabolomics results, oleanane-type saponins predominantly accumulate in rhizomes (ZA), dammarane-type saponins were primarily concentrated in leaves (ZC), representing the most abundant triterpenoid saponins exhibiting tissue-specific differences between these two tissues. Consequently, rhizomes (ZA) and leaves (ZC) were selected as subjects for subsequent transcriptomic analysis to investigate the regulatory mechanisms governing saponin biosynthesis.

### 3.2 Transcriptomics analysis

#### 3.2.1 Filtering of sequencing data

In this study, transcriptome sequencing was performed on the rhizomes (ZA) and leaves (ZC) of *P. japonicus* var. *major*. The raw sequencing data were subjected to quality filtering to obtain valid reads (Table 2). After quality filtering, clean bases of at least 6.2 Gb were generated for all samples, with an error rate of 0.03 %. These results validate the high quality and reliability of the sequencing data, which are suitable for subsequent bioinformatic analyses.

**Table 2:** Filtering of sequencing data.

Sample	Raw reads	Clean reads	Clean base (Gb)	Error rate (%)	Q20 (%)	Q30 (%)	GC content (%)
ZA-1	42,702,172	41,612,752	6.24	0.03	97.76	92.89	43.59
ZA-2	48,162,632	47,174,318	7.08	0.03	97.71	92.73	43.58
ZA-3	45,858,852	45,043,264	6.76	0.03	97.72	92.77	43.63
ZC-1	44,227,250	43,214,856	6.48	0.03	97.76	92.87	43.45
ZC-2	44,257,588	43,199,542	6.48	0.03	97.75	92.83	43.33
ZC-3	50,054,252	48,909,320	7.34	0.03	97.75	92.84	43.35

#### 3.2.2 Transcriptome assembly

Filtered transcriptome data were assembled with Trinity, and the generated transcripts served as reference sequences for subsequent analyses. Following hierarchical clustering by Corset, the most extended cluster sequences were defined as Unigenes for subsequent analysis (Table 3).

**Table 3:** Assembly result statistics table.

Type	Number	Mean length	N50	N90	Total bases
Transcript	244,513	997	1,884	357	243,884,592
Unigene	164,219	1,356	2,060	609	222,611,953

**Figure 9:**
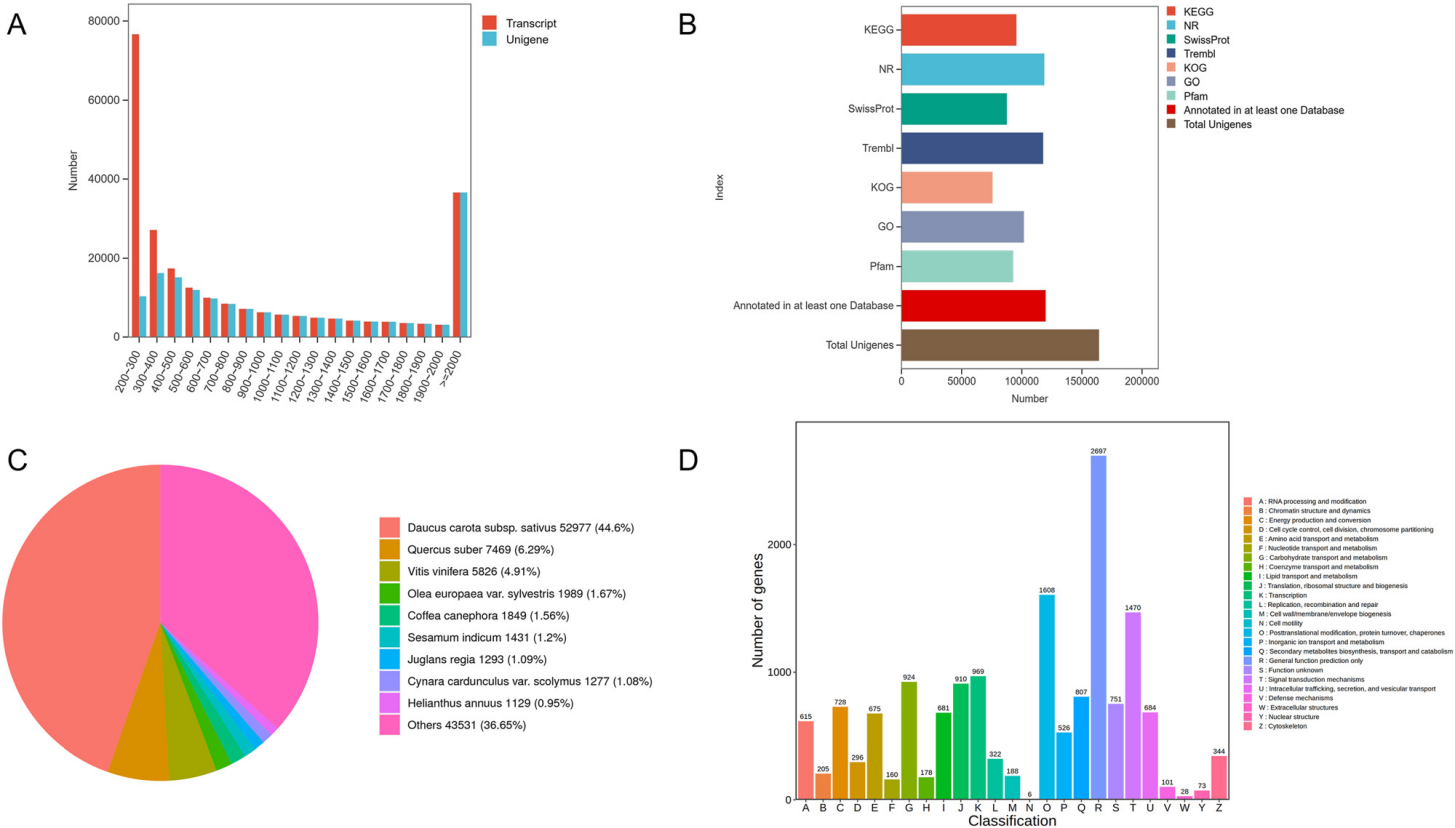
Functional annotation of Unigenes (A) Sequence length distribution. The *x*-axis represents the length of the transcript and Unigene, the *y*-axis represents the number of sequences. (B) Unigenes annotation statistics. The *x*-axis represents the annotation status of each database, while the *y*-axis represents different types of databases. (C) Nr database annotation. (D) KOG database annotation. The *x*-axis represents the various categories of KOG, while the *y*-axis represents the number of genes.

Length distribution analysis revealed 41,690 Unigene sequences (<500 nt, 25.39 %), 43,457 sequences (500–1,000 nt, 26.46 %), 42,492 sequences (1,000–2,000 nt, 25.88 %), and 36,580 sequences (>2,000 nt, 22.28 %). The above results show that the integrity of the assembly results is high (Figure 9A).

#### 3.2.3 Gene function annotation

The 164,219 Unigene sequences were compared with KEGG, NR, Swiss-Prot, GO, KOG, and Trembl databases using BLAST software under the conditions of Evalue 1e-5 or less, identity of 30 % or more, and sequence coverage of 30 % or more. After predicting the amino acid sequence of Unigene use HMMER software to compare with Pfam database to obtain the annotation information of Unigene. Among the 164,219 Unigenes obtained, 95,514 (58.16 %) were annotated in KEGG, including 118,771 (72.32 %) in Nr, 87,614 (53.35 %) in SwissProt, 117,746 (71.70 %) in Trembl, 75,655 (46.07 %) in KOG, 101,779 (61.98 %) in GO, and 92,823 (56.52 %) in Pfam. In total, 119,830 Unigenes (72.97 %) were successfully annotated in at least one database (Figure 9B).

#### 3.2.4 Nr database annotation

The *P. japonicus* var. *major* gene sequences were comparatively annotated in the Nr database, and the proportion of closely related species was as follows: 44.60 % of the Unigene sequences were similar to *Daucus carota subsp. sativus*; 6.29 % of the Unigene were similar to *Quercus suber*; 4.91 % of the Unigene were similar to *Vitis vinifera*; 1.67 % Unigene was similar to *Olea europaea var. sylvestris*; 1.56 % Unigene was similar to *Coffea canephora*; 1.20 % Unigene was similar to *Sesamum indicum*; 1.09 % of the Unigene was similar to *Juglans regia*; 1.08 % Unigene was similar to *Cynara cardunculus var. scolymus*; 0.95 % Unigene was similar to *Helianthus annuus*; 0.86 % Unigene was similar to *Lactuca sativa* similar; 36.65 % of Unigene were similar to other species (Figure 9C).

#### 3.2.5 Differentially expressed genes analysis

After DESeq2 analysis, 26,988 DEGs were identified in rhizomes (ZA) and leaves (ZC), of which 12,357 were highly expressed in rhizomes (ZA) and 14,631 in leaves (ZC).

#### 3.2.6 KOG database annotation

DEGs were annotated to the KOG database, and Unigenes were assigned to 25 functional groups (Figure 9D). Generic function prediction only was the largest share of the 25 functional groups totaling 2,697 DEGs, 16.91 %; followed by Posttranslational modification, protein turnover, and chaperones totaling 1,608 DEGs, 10.08 %; then Signal transduction mechanisms totaling 1,470 DEGs, 9.22 %; Cell cycle control, cell division, chromosome partitioning totaling 296 DEGs, 1.86 %; and Cell wall/membrane/envelope biogenesis totaling 188 DEGs, 1.18 %; Cell motility totaling 6 DEGs, 0.04 %; Intracellular trafficking, secretion, and vesicular transport totaling 684 DEGs, 4.29 %; Defense mechanisms totaling 101 DEGs, 0.63 %; Extracellular structures totaling 28 DEGs, 0.18 %; Nuclear structure totaling 73 DEGs, 0.46 %; Cytoskeleton totaling 344 DEGs, 2.16 %; RNA processing and modification totaling 615 DEGs, 3.86 %; Chromatin structure and dynamics totaling 205 DEGs, 1.29 %; Translation, ribosomal structure and biogenesis totaling 910 DEGs, 5.71 %; and Transcription totaling 969 DEGs, 6.08 %; Replication, recombination and repair totaling 322 DEGs, 2.02 %; Energy production and conversion totaling 728 DEGs, 4.57 %; Amino acid transport and metabolism totaling 675 DEGs, 4.23 %; Nucleotide transport and metabolism totaling 160 DEGs, 1.00 %; Carbohydrate transport and metabolism totaling 924 DEGs, 5.79 %; Coenzyme transport and metabolism totaled 178 DEGs, 1.12 %; Lipid transport and metabolism totaled 681 DEGs, 4.27 %; Inorganic ion transport and metabolism totaled 526 DEGs, 3.30 %; Secondary metabolites biosynthesis, transport, and catabolism totaled 807 DEGs, 5.06 %, as well as Functions unknown totaled 751 DEGs, 4.71 %.

#### 3.2.7 GO enrichment analysis

**Figure 10:**
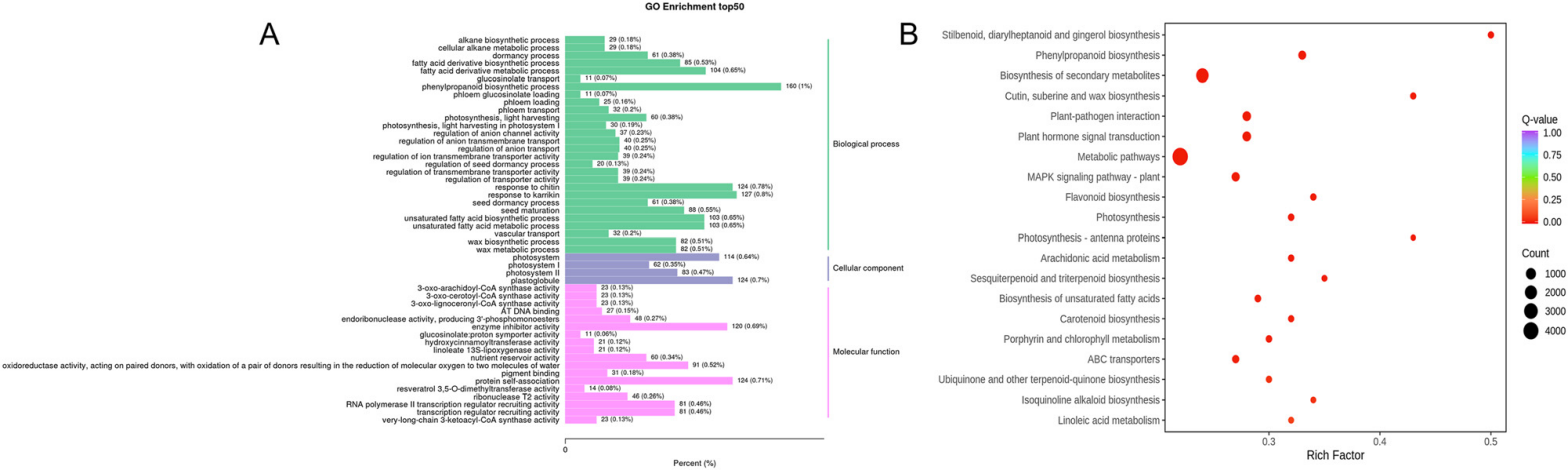
GO and KEGG enrichment analyses of DEGs. (A) GO enrichment analysis of DEGs. Select the 50 GO-terms with the lowest *q*-values from the enrichment analysis results and plot a bar chart of the enriched terms. The *x*-axis represents the proportion of annotated genes assigned to each GO-term relative to the total number of annotated genes, and the *y*-axis represents the names of the GO-terms. The labels on the right side of the graph indicate the category to which the GO-term belongs. (B) KEGG enrichment bubble plot of DEGs between rhizomes (ZA) and leaves (ZC).

The functions in the GO database can be classified into three major categories: Biological process, Cellular component, and Molecular function. GO enrichment analysis indicates that within the Biological process category, the highly enriched terms include phenylpropanoid biosynthetic process, response to karrikin, and response to chitin. Within the Cellular component category, highly enriched terms include plastoglobule and photosystem. In the Molecular function category, highly enriched terms include protein self-association and enzyme inhibitor activity (Figure 10A).

#### 3.2.8 KEGG database annotation and KEGG enrichment analysis

A total of 9,640 DEGs were mapped to 142 KEGG metabolic pathways, which can be divided into six categories: Cellular processes, Environmental information processing, Genetic information processing, Human Diseases, Metabolism and Organismal systems (Supplementary Table 11). These genes were involved in pathways associated with stilbenoid, diarylheptanoid and gingerol biosynthesis, phenylpropanoid biosynthesis, cutin, suberine and wax biosynthesis, photosynthesiss-antenna proteins, sesquiterpenoid and triterpenoid biosynthesis, ubiquinone and other terpenoid-quinone biosynthesis, plant-pathogen interaction, plant hormone signal transduction, photosynthesis, flavonoid biosynthesis, and MAPK signaling pathway- plant (Figure 10B).

#### 3.2.9 Key enzyme genes associated with triterpenoid saponin biosynthesis

A total of 87 key enzyme genes were annotated to pathways, with genes associated with the MVA pathway primarily expressed in rhizomes (ZA), while those linked to the MEP pathway were mainly expressed in leaves (ZC). Analyzing these differential genes can provide clearer information on the biosynthesis of triterpenoid saponins (Table 4).

**Table 4:** Key enzyme genes associated with triterpenoid saponin biosynthesis.

Name	Up	Down	Name	Up	Down
AACT	0	2	MECPs	2	0
HMGS	0	2	HDS	0	4
HMGR	0	4	HDR	2	5
MVK	0	6	IDI	0	1
PMK	1	1	β-AS	3	4
MVD	1	0	SS	0	3
DXS	6	5	SQE	5	2
DXR	1	1	FPPS	4	6
MCT	0	4	GGPPS	8	4

## 4 Discussion

*P. japonicus* var. *major* is a precious medicinal herb. Its bioactive constituents are saponins, which exert pharmacological effects including cardiovascular protection and anti-tumor activity [22]. This study found that the rare saponin, ginsenoside Rd, is enriched in the leaves of the ginseng species *P. japonicus* var. *major*. This compound exhibits multiple pharmacological activities, including antioxidant effects and protection of the blood-brain barrier [23]. This study established the metabolome profile of *P. japonicus* var. *major* through metabolomic analysis of different plant tissues and transcriptomic sequencing of rhizomes (ZA) and leaves (ZC). It identified many triterpenoid saponin biosynthesis genes, laying the foundation for in-depth exploration of the biosynthesis pathways of these genes and for the optimization of germplasm resources of *P. japonicus* var. *major*.

### 4.1 Metabolomics analysis

In this study, we employed widely targeted metabolomics technology to analyse five different tissues of *P. japonicus* var. *major*, and 384 metabolites were identified. PCA and OPLS-DA analysis demonstrated substantial differences in the types of metabolites present across the various tissues of *P. japonicus* var. *major*.

The accumulation of upstream carbon metabolism in triterpenoid saponin biosynthesis across different tissues demonstrates that carbon metabolism provides the essential energy for plant growth, development, and vital functions. Sugar metabolism, glycolysis, the TCA cycle, and shikimate-phenylalanine metabolism – all fall within the broad scope of carbon metabolism [24]. d-glucose-6-phosphate, which accumulated significantly in rhizomes (ZA) and internodes (ZB), serves as the initial metabolite of glycolysis, the starting substrate of the pentose phosphate pathway, and the terminal product of gluconeogenesis. These three interconnected pathways constitute core components of glucose metabolism, supporting plant growth by supplying energy and diverse biosynthetic precursors. The marked accumulation of d-glucose-6-phosphate indicates that rhizomes (ZA) and internodes (ZB) have a high energy and intermediate metabolite demand to sustain multiple downstream biosynthetic processes. D-(+)-sucrose mainly accumulates in the rhizomes (ZA) and internodes (ZB). It is the transport sugar in plants and can enter glycolysis and the TCA cycle to produce ATP and NADH [25]. Acetyl-CoA in the TCA cycle is the first step in the common MVA pathway for terpenoid biosynthesis. Two TCA cycle intermediates, succinic acid and citric acid, showed distinct tissue distribution: succinic acid was abundant in leaves (ZC), whereas citric acid accumulated predominantly in rhizomes (ZA) and internodes (ZB).

Analysis of the terpenoids clustering heatmap indicates that all five tissues of *P. japonicus* var. *major* contain triterpenoid saponins, though the specific saponin types present differ among tissues. The rhizomes (ZA) and internodes (ZB) primarily enriched oleanane-type triterpenoid saponins, such as chikusetsusaponin IVa and chikusetsusaponin V; Dammarane-type saponins were predominantly concentrated in leaves (ZC), while a few PPT-type triterpenoid saponins, such as notoginsenoside R1, were enriched in rhizomes (ZA). This is in sharp contrast to the distribution of saponins in other Araliaceae plants like *P. ginseng*, *P. notoginseng*, and American ginseng, which mainly contain dammarane-type saponins [26]. This provides a new perspective for the study of the biosynthetic pathway of triterpenoid saponins.

### 4.2 Transcriptome sequencing and differential gene function annotation

This study conducted transcriptome sequencing on the rhizomes (ZA) and leaves (ZC) of *P. japonicus* var. *major*, yielding a total of 164,219 unigenes. Differential gene analysis revealed 26,988 DEGs between the two tissues, with 12,357 highly expressed in rhizomes (ZA) and 14,631 in leaves (ZC).

To elucidate the triterpenoid saponin biosynthesis mechanism in *P. japonicus* var. *major*, a total of 87 key enzyme genes associated with triterpenoid saponin biosynthesis pathway were identified. Among these, genes *AACT*, *HMGS*, *HMGR*, and *MVK* from the MVA pathway; genes *MCT*, *HDS*, and *HDR* from the MEP pathway; IDI, the key enzyme supporting terpenoid skeleton construction; and genes *SS* and *FPPS* from the 2,3-oxidosqualene pathway were all highly expressed in rhizomes (ZA). The key genes *DXS* and *MECPs* in the MEP pathway, along with *SQE* in the 2,3-oxidosqualene pathway, were predominantly highly expressed in leaves (ZC). The expression patterns of the two key enzymes identified in this study (MECPs, SQE) align with findings reported by He et al. [27] in *Hylomecon japonica*. β-AS is the key enzyme in the synthesis of oleanane-type triterpenoid saponins, catalyzing the conversion of 2,3-oxidosqualene into β-amyrin, which undergoes modifications by glycosyltransferases to form oleanane-type triterpenoid saponins ultimately [28]. This study detected high expression of the *β-* *AS* key gene in rhizomes (ZA), indicating that oleanane-type triterpenoid saponins in *P. japonicus* var. *major* are primarily synthesised in the rhizomes (ZA). This finding aligns with metabolomics results, further substantiating that oleanane-type triterpenoid saponins predominantly accumulate in rhizomes (ZA).

The *MVD* gene in the MVA pathway, the *DXS *and *MECPs* genes in the MEP pathway, and the *SQE* gene in the 2,3-oxidosqualene pathway are primarily highly expressed in leaves (ZC). The MVA and MEP pathways constitute the two core precursor pathways for triterpenoid saponin biosynthesis. Within the MVA pathway, the *MVD* gene catalyses the synthesis of IPP, providing the fundamental precursor for the triterpenoid saponin skeleton [29]. The high expression of *DXS* and *MECPs*, as key enzyme genes in the MEP pathway, indicates increased synthesis of terpenoid precursors in leaves (ZC) [30]. SQE catalyzes the conversion of squalene to 2,3-oxidosqualene, and the key *SQE* gene provides precursor substances for the biosynthesis of triterpenoid saponins. The *SQE* gene is highly expressed in leaves (ZC), consistent with the findings of He et al. [27], suggesting that the high expression of the key *SQE* enzyme gene may provide the necessary material support for the synthesis of terpenoid derivatives in leaves (ZC), thereby participating in and regulating the triterpenoid saponin biosynthesis [31]. The *PMK* gene in the MVA pathway and the *DXR* gene in the MEP pathway did not exhibit significant tissue-specific differences in rhizomes (ZA) and leaves (ZC). However, Zhang et al. [32] found that both genes were highly expressed in flowers of *P.* *notoginseng*; thus, the expression patterns of genes involved in terpenoid precursor synthesis may vary among different *Panax* species.

This study identified 87 key enzyme genes involved in triterpenoid saponin biosynthesis, confirming that the oleanane-type triterpenoid saponins of *P. japonicus* var. *major* exhibit rhizome-specific synthesis and accumulation patterns. Leaves primarily contribute to terpenoid precursor synthesis and coordinate whole-plant metabolism. This tissue-specific regulatory model provides a foundation for research into the medicinal resources and quality improvement of *P. japonicus* var. *major*.

## 5 Conclusions

This study employed a widely targeted metabolomics technology to analyse rhizomes, internodes, leaves, petioles, and stems from *P. japonicus* var. *major*. Metabolomic results revealed distinct patterns of metabolite composition across different tissues. The primary metabolites associated with energy requirements for triterpenoid saponin biosynthesis are predominantly concentrated in the rhizomes, internodes, and leaves. Cluster analysis of triterpenoid metabolites further indicated that oleanane-type triterpenoid saponins predominantly accumulated in rhizomes, whereas dammarane-type triterpenoid saponins were enriched in leaves. Transcriptome sequencing of rhizomes and leaves identified 87 key enzyme genes associated with triterpenoid saponin biosynthesis. Combined with metabolomic analysis, it was confirmed that oleanane-type triterpenoid saponins exhibit specific accumulation characteristics in the rhizomes. This study preliminarily elucidates that oleanane-type triterpenoid saponins are mainly synthesized and specifically accumulated in the rhizome of *P. japonicus* var. *major*, providing a theoretical foundation for further investigations into the biosynthesis and accumulation mechanisms of these compounds.

In order to study the biosynthesis and regulatory mechanism of the active ingredients of *P. japonicus* var. *major*, the next step could be to supplement the analysis of transcription factors and screen out regulatory genes. According to the validation results, the functional verification of the candidate genes was carried out to further analyze the characteristics of the biosynthetic pathway of ginsenosides in *P. japonicus* var. *major*, so as to lay a foundation for better cultivation and resource utilization of *P. japonicus* var. *major*.

## Supplementary Material

Supplementary Material
