# Supplementary material for: Study on the tissue-specific distribution of triterpenoid saponins in Panax japonicus var. major based on metabolomics and transcriptomics
Source: Open Life Sci. 2026 Apr 8;21(1):20251300. doi: 10.1515/biol-2025-1300 (PMC13065427; doi:10.1515/biol-2025-1300)
Supplement: Supplementary file 1 — Supplementary Material [file j_biol-2025-1300_suppl_001.docx]

Supplementary Table 1: Accumulation of triterpenoid saponins in ZA and ZB

| Index | Formula | Compounds | VIP | | Fold_Change | Type |
| --- | --- | --- | --- | --- | --- | --- |
| Lmzn003299 | C54H92O23 | Ginsenoside Rb1 | | 1.12 | 3.62 | up |
| Lmzn005163 | C50H84O19 | 6″-Acetyl-ginsenoside Rd | | 1.02 | 2.26 | up |
| Lmzp004417 | C36H62O9 | Ginsenoside Rf1 | | 1.12 | 3.60 | up |
| Lmzp004450 | C42H72O15 | Majoroside R1 | | 1.12 | 21.16 | up |
| Lmzp004643 | C41H70O14 | Majoroside R2 | | 1.12 | 18.35 | up |
| Lmzp004896 | C36H62O10 | (24S)-Pseudo-ginsenoside RT4 | | 1.12 | 27.98 | up |
| Lmzp005197 | C42H70O12 | Ginsenoside Rg5 | | 1.11 | 2.65 | up |
| mws4022 | C42H72O13 | 20(S)-Ginsenoside Rg3 | | 1.12 | 5387.33 | up |
| mws4023 | C42H72O13 | Ginsenosides Rg2 | | 1.09 | 3.02 | up |
| mws4027 | C36H62O10 | Pseudoginsenoside RT5 | | 1.12 | 28.27 | up |
| mws4035 | C42H72O13 | Ginsenoside F2 | | 1.12 | 8.67 | up |
| pmn001505 | C41H64O13 | Oleanolic acid-3-O-β-D-pyran xylose(1→3)-β-D-pyran glucuronide | | 1.12 | 3.05 | up |

Supplementary Table 2: Accumulation of triterpenoid saponins in ZA and ZC

| Index | Formula | Compounds | VIP | Fold_Change | Type |
| --- | --- | --- | --- | --- | --- |
| Lmzn003299 | C54H92O23 | Ginsenoside Rb1 | 1.06 | 0.12 | down |
| Lmzn003820 | C44H74O15 | Yesanchinosides D | 1.05 | 2.78 | up |
| Lmzn004178 | C48H82O19 | Majoroside F1 | 1.07 | 7.86 | up |
| Lmzn004494 | C56H94O24 | Quinquenoside R1 | 1.05 | 0.38 | down |
| Lmzn004497 | C58H98O26 | Notoginsenoside Fc | 1.06 | 0.49 | down |
| Lmzn004691 | C48H76O19 | Chikusetsusaponin V | 1.07 | 0.03 | down |
| Lmzn004931 | C42H66O14 | Zingibroside R1 | 1.07 | 0.00 | down |
| Lmzn004933 | C43H68O14 | Chikusetsusaponin IV methyl ester | 1.07 | 0.00 | down |
| Lmzn005163 | C50H84O19 | 6″-Acetyl-ginsenoside Rd | 1.06 | 3.34 | up |
| Lmzp004417 | C36H62O9 | Ginsenoside Rf1 | 1.07 | 9.25 | up |
| Lmzp004643 | C41H70O14 | Majoroside R2 | 1.07 | 16.10 | up |
| Lmzp004896 | C36H62O10 | (24S)-Pseudo-ginsenoside RT4 | 1.07 | 23.56 | up |
| Lmzp005197 | C42H70O12 | Ginsenoside Rg5 | 1.07 | 20.91 | up |
| Lmzp005427 | C48H82O18 | Ginsenoside Rd | 1.07 | 11.64 | up |
| Lmzp006292 | C42H66O14 | Chikusetsusaponin IVa | 1.07 | 0.00 | down |
| mws1553 | C42H72O14 | Ginsenoside Rg1 | 1.06 | 4.09 | up |
| mws1592 | C48H76O19 | Ginsenoside Ro | 1.07 | 0.03 | down |
| mws1721 | C47H80O18 | Notoginsenoside R1 | 1.06 | 0.19 | down |
| mws4022 | C42H72O13 | 20(S)-Ginsenoside Rg3 | 1.07 | 128507.41 | up |
| mws4023 | C42H72O13 | Ginsenosides Rg2 | 1.07 | 41.44 | up |
| mws4027 | C36H62O10 | Pseudoginsenoside RT5 | 1.07 | 21.83 | up |
| mws4031 | C42H70O12 | Ginsenoside Rk1 | 1.07 | 5.63 | up |
| mws4035 | C42H72O13 | Ginsenoside F2 | 1.07 | 117.28 | up |
| mws4036 | C36H62O9 | 20(S)-Ginsenoside Rh1 | 1.07 | 36.55 | up |
| pmn001505 | C41H64O13 | Oleanolic acid-3-O-β-D-pyran xylose(1→3)-β-D-pyran glucuronide | 1.07 | 0.00 | down |
| pmn001506 | C36H58O8 | Oleanolic acid 2-O-β-D-glucopyranoside | 1.07 | 0.00 | down |

Supplementary Table 3: Accumulation of triterpenoid saponins in ZA and ZD

| Index | Formula | Compounds | VIP | Fold_Change | Type |
| --- | --- | --- | --- | --- | --- |
| Lmzn003820 | C44H74O15 | Yesanchinosides D | 1.03 | 2.50 | up |
| Lmzn004178 | C48H82O19 | Majoroside F1 | 1.06 | 2.85 | up |
| Lmzn004494 | C56H94O24 | Quinquenoside R1 | 1.03 | 0.38 | down |
| Lmzn004497 | C58H98O26 | Notoginsenoside Fc | 1.04 | 0.38 | down |
| Lmzn004691 | C48H76O19 | Chikusetsusaponin V | 1.06 | 0.09 | down |
| Lmzn004931 | C42H66O14 | Zingibroside R1 | 1.07 | 0.10 | down |
| Lmzn004933 | C43H68O14 | Chikusetsusaponin IV methyl ester | 1.07 | 0.00 | down |
| Lmzp004417 | C36H62O9 | Ginsenoside Rf1 | 1.06 | 0.47 | down |
| Lmzp004450 | C42H72O15 | Majoroside R1 | 1.06 | 9.70 | up |
| Lmzp004643 | C41H70O14 | Majoroside R2 | 1.07 | 36.13 | up |
| Lmzp004896 | C36H62O10 | (24S)-Pseudo-ginsenoside RT4 | 1.07 | 51.48 | up |
| Lmzp005197 | C42H70O12 | Ginsenoside Rg5 | 1.04 | 2.60 | up |
| Lmzp006292 | C42H66O14 | Chikusetsusaponin IVa | 1.06 | 0.14 | down |
| mws1592 | C48H76O19 | Ginsenoside Ro | 1.07 | 0.08 | down |
| mws1666 | C48H82O18 | Ginsenoside Re | 1.04 | 0.46 | down |
| mws1721 | C47H80O18 | Notoginsenoside R1 | 1.06 | 0.10 | down |
| mws4022 | C42H72O13 | 20(S)-Ginsenoside Rg3 | 1.07 | 2440.48 | up |
| mws4023 | C42H72O13 | Ginsenosides Rg2 | 1.06 | 24.82 | up |
| mws4027 | C36H62O10 | Pseudoginsenoside RT5 | 1.07 | 49.43 | up |
| mws4031 | C42H70O12 | Ginsenoside Rk1 | 1.05 | 0.20 | down |
| mws4035 | C42H72O13 | Ginsenoside F2 | 1.06 | 5.04 | up |
| mws4036 | C36H62O9 | 20(S)-Ginsenoside Rh1 | 1.05 | 3.20 | up |
| pmn001505 | C41H64O13 | Oleanolic acid-3-O-β-D-pyran xylose(1→3)-β-D-pyran glucuronide | 1.07 | 0.00 | down |
| pmn001506 | C36H58O8 | Oleanolic acid 2-O-β-D-glucopyranoside | 1.06 | 0.01 | down |

Supplementary Table 4: Accumulation of triterpenoid saponins in ZA and ZE

| Index | Formula | Compounds | VIP | Fold_Change | Type |
| --- | --- | --- | --- | --- | --- |
| Lmzn003940 | C48H80O19 | Notoginsenoside G | 1.05 | 0.34 | down |
| Lmzn004494 | C56H94O24 | Quinquenoside R1 | 1.05 | 0.21 | down |
| Lmzn004497 | C58H98O26 | Notoginsenoside Fc | 1.05 | 0.21 | down |
| Lmzn004691 | C48H76O19 | Chikusetsusaponin V | 1.06 | 0.12 | down |
| Lmzn004931 | C42H66O14 | Zingibroside R1 | 1.06 | 0.23 | down |
| Lmzn004933 | C43H68O14 | Chikusetsusaponin IV methyl ester | 1.06 | 0.00 | down |
| Lmzn005163 | C50H84O19 | 6″-Acetyl-ginsenoside Rd | 1.05 | 0.33 | down |
| Lmzp004417 | C36H62O9 | Ginsenoside Rf1 | 1.06 | 0.29 | down |
| Lmzp004450 | C42H72O15 | Majoroside R1 | 1.06 | 4.27 | up |
| Lmzp004643 | C41H70O14 | Majoroside R2 | 1.06 | 21.10 | up |
| Lmzp004896 | C36H6O10 | (24S)-Pseudo-ginsenoside RT4 | 1.06 | 33.23 | up |
| Lmzp006292 | C42H66O14 | Chikusetsusaponin IVa | 1.06 | 0.22 | down |
| mws1553 | C42H72O14 | Ginsenoside Rg1 | 1.06 | 0.30 | down |
| mws1592 | C48H76O19 | Ginsenoside Ro | 1.06 | 0.11 | down |
| mws1665 | C42H72O14 | Ginsenoside Rf | 1.03 | 0.40 | down |
| mws1666 | C48H82O18 | Ginsenoside Re | 1.05 | 0.34 | down |
| mws1721 | C47H80O18 | Notoginsenoside R1 | 1.06 | 0.04 | down |
| mws4022 | C42H72O13 | 20(S)-Ginsenoside Rg3 | 1.06 | 254.53 | up |
| mws4023 | C42H72O13 | Ginsenosides Rg2 | 1.05 | 7.30 | up |
| mws4027 | C36H62O10 | Pseudoginsenoside RT5 | 1.06 | 30.26 | up |
| mws4031 | C42H70O12 | Ginsenoside Rk1 | 1.06 | 0.00 | down |
| mws4035 | C42H72O13 | Ginsenoside F2 | 1.06 | 4.13 | up |
| mws4036 | C36H62O9 | 20(S)-Ginsenoside Rh1 | 1.06 | 0.00 | down |
| pmn001505 | C41H64O13 | Oleanolic acid-3-O-β-D-pyran xylose(1→3)-β-D-pyran glucuronide | 1.06 | 0.11 | down |
| pmn001506 | C36H58O8 | Oleanolic acid 2-O-β-D-glucopyranoside | 1.06 | 0.22 | down |

Supplementary Table 5: Accumulation of triterpenoid saponins in ZB and ZC

| Index | Formula | Compounds | VIP | Fold_Change | Type |
| --- | --- | --- | --- | --- | --- |
| Lmzn003299 | C54H92O23 | Ginsenoside Rb1 | 1.05 | 0.03 | down |
| Lmzn003820 | C44H74O15 | Yesanchinosides D | 1.04 | 3.27 | up |
| Lmzn004178 | C48H82O19 | Majoroside F1 | 1.06 | 6.75 | up |
| Lmzn004494 | C56H94O24 | Quinquenoside R1 | 1.04 | 0.34 | down |
| Lmzn004497 | C58H98O26 | Notoginsenoside Fc | 1.05 | 0.39 | down |
| Lmzn004691 | C48H76O19 | Chikusetsusaponin V | 1.06 | 0.04 | down |
| Lmzn004931 | C42H66O14 | Zingibroside R1 | 1.06 | 0.00 | down |
| Lmzn004933 | C43H68O14 | Chikusetsusaponin IV methyl ester | 1.06 | 0.00 | down |
| Lmzp004417 | C36H62O9 | Ginsenoside Rf1 | 1.05 | 2.57 | up |
| Lmzp004450 | C42H72O15 | Majoroside R1 | 1.06 | 0.09 | down |
| Lmzp005197 | C42H70O12 | Ginsenoside Rg5 | 1.06 | 7.88 | up |
| Lmzp005427 | C48H82O18 | Ginsenoside Rd | 1.05 | 6.82 | up |
| Lmzp006292 | C42H66O14 | Chikusetsusaponin IVa | 1.06 | 0.00 | down |
| mws1553 | C42H72O14 | Ginsenoside Rg1 | 1.05 | 4.90 | up |
| mws1592 | C48H76O19 | Ginsenoside Ro | 1.06 | 0.03 | down |
| mws1721 | C47H80O18 | Notoginsenoside R1 | 1.04 | 0.21 | down |
| mws4022 | C42H72O13 | 20(S)-Ginsenoside Rg3 | 1.06 | 23.85 | up |
| mws4023 | C42H72O13 | Ginsenosides Rg2 | 1.06 | 13.72 | up |
| mws4031 | C42H70O12 | Ginsenoside Rk1 | 1.05 | 3.62 | up |
| mws4035 | C42H72O13 | Ginsenoside F2 | 1.06 | 13.53 | up |
| mws4036 | C36H62O9 | 20(S)-Ginsenoside Rh1 | 1.06 | 34.46 | up |
| pmn001505 | C41H64O13 | Oleanolic acid-3-O-β-D-pyran xylose(1→3)-β-D-pyran glucuronide | 1.06 | 0.00 | down |
| pmn001506 | C36H58O8 | Oleanolic acid 2-O-β-D-glucopyranoside | 1.06 | 0.00 | down |

Supplementary Table 6: Accumulation of triterpenoid saponins in ZB and ZD

| Index | Formula | Compounds | VIP | Fold_Change | Type |
| --- | --- | --- | --- | --- | --- |
| Lmzn003299 | C54H92O23 | Ginsenoside Rb1 | 1.06 | 0.21 | down |
| Lmzn003820 | C44H74O15 | Yesanchinosides D | 1.04 | 2.94 | up |
| Lmzn004178 | C48H82O19 | Majoroside F1 | 1.06 | 2.45 | up |
| Lmzn004494 | C56H94O24 | Quinquenoside R1 | 1.04 | 0.33 | down |
| Lmzn004497 | C58H98O26 | Notoginsenoside Fc | 1.05 | 0.30 | down |
| Lmzn004691 | C48H76O19 | Chikusetsusaponin V | 1.06 | 0.11 | down |
| Lmzn004931 | C42H66O14 | Zingibroside R1 | 1.07 | 0.10 | down |
| Lmzn004933 | C43H68O14 | Chikusetsusaponin IV methyl ester | 1.06 | 0.00 | down |
| Lmzn005163 | C50H84O19 | 6″-Acetyl-ginsenoside Rd | 1.03 | 0.25 | down |
| Lmzp004417 | C36H62O9 | Ginsenoside Rf1 | 1.06 | 0.13 | down |
| Lmzp004450 | C42H72O15 | Majoroside R1 | 1.05 | 0.46 | down |
| Lmzp006292 | C42H66O14 | Chikusetsusaponin IVa | 1.05 | 0.15 | down |
| mws1592 | C48H76O19 | Ginsenoside Ro | 1.06 | 0.09 | down |
| mws1666 | C48H82O18 | Ginsenoside Re | 1.05 | 0.45 | down |
| mws1721 | C47H80O18 | Notoginsenoside R1 | 1.06 | 0.11 | down |
| mws4022 | C42H72O13 | 20(S)-Ginsenoside Rg3 | 1.06 | 0.45 | down |
| mws4023 | C42H72O13 | Ginsenosides Rg2 | 1.06 | 8.21 | up |
| mws4031 | C42H70O12 | Ginsenoside Rk1 | 1.05 | 0.13 | down |
| mws4036 | C36H62O9 | 20(S)-Ginsenoside Rh1 | 1.06 | 3.02 | up |
| pmn001505 | C41H64O13 | Oleanolic acid-3-O-β-D-pyran xylose(1→3)-β-D-pyran glucuronide | 1.07 | 0.00 | down |
| pmn001506 | C36H58O8 | Oleanolic acid 2-O-β-D-glucopyranoside | 1.06 | 0.01 | down |

Supplementary Table 7: Accumulation of triterpenoid saponins in ZB and ZE

| Index | Formula | Compounds | VIP | Fold_Change | Type |
| --- | --- | --- | --- | --- | --- |
| Lmzn003940 | C48H80O19 | Notoginsenoside G | 1.06 | 0.27 | down |
| Lmzn004494 | C56H94O24 | Quinquenoside R1 | 1.06 | 0.19 | down |
| Lmzn004497 | C58H98O26 | Notoginsenoside Fc | 1.06 | 0.16 | down |
| Lmzn004691 | C48H76O19 | Chikusetsusaponin V | 1.07 | 0.13 | down |
| Lmzn004931 | C42H66O14 | Zingibroside R1 | 1.07 | 0.24 | down |
| Lmzn004933 | C43H68O14 | Chikusetsusaponin IV methyl ester | 1.07 | 0.00 | down |
| Lmzn005163 | C50H84O19 | 6″-Acetyl-ginsenoside Rd | 1.05 | 0.15 | down |
| Lmzp004417 | C36H62O9 | Ginsenoside Rf1 | 1.07 | 0.08 | down |
| Lmzp004450 | C42H72O15 | Majoroside R1 | 1.06 | 0.20 | down |
| Lmzp006292 | C42H66O14 | Chikusetsusaponin IVa | 1.05 | 0.23 | down |
| mws1553 | C42H72O14 | Ginsenoside Rg1 | 1.07 | 0.36 | down |
| mws1592 | C48H76O19 | Ginsenoside Ro | 1.07 | 0.11 | down |
| mws1665 | C42H72O14 | Ginsenoside Rf | 1.07 | 0.24 | down |
| mws1666 | C48H82O18 | Ginsenoside Re | 1.06 | 0.34 | down |
| mws1721 | C47H80O18 | Notoginsenoside R1 | 1.07 | 0.05 | down |
| mws4022 | C42H72O13 | 20(S)-Ginsenoside Rg3 | 1.07 | 0.05 | down |
| mws4023 | C42H72O13 | Ginsenosides Rg2 | 1.06 | 2.42 | up |
| mws4031 | C42H70O12 | Ginsenoside Rk1 | 1.07 | 0.00 | down |
| mws4035 | C42H72O13 | Ginsenoside F2 | 1.05 | 0.48 | down |
| mws4036 | C36H62O9 | 20(S)-Ginsenoside Rh1 | 1.07 | 0.00 | down |
| pmn001505 | C41H64O13 | Oleanolic acid-3-O-β-D-pyran xylose(1→3)-β-D-pyran glucuronide | 1.07 | 0.04 | down |
| pmn001506 | C36H58O8 | Oleanolic acid 2-O-β-D-glucopyranoside | 1.07 | 0.03 | down |

Supplementary Table 8: Accumulation of triterpenoid saponins in ZC and ZD

| Index | Formula | Compounds | VIP | Fold_Change | Type |
| --- | --- | --- | --- | --- | --- |
| Lmzn003299 | C54H92O23 | Ginsenoside Rb1 | 1.06 | 6.43 | up |
| Lmzn004178 | C48H82O19 | Majoroside F1 | 1.07 | 0.36 | down |
| Lmzn004691 | C48H76O19 | Chikusetsusaponin V | 1.05 | 2.96 | up |
| Lmzn004931 | C42H66O14 | Zingibroside R1 | 1.08 | 39.35 | up |
| Lmzn005163 | C50H84O19 | 6″-Acetyl-ginsenoside Rd | 1.07 | 0.17 | down |
| Lmzp004417 | C36H62O9 | Ginsenoside Rf1 | 1.08 | 0.05 | down |
| Lmzp004450 | C42H72O15 | Majoroside R1 | 1.07 | 4.88 | up |
| Lmzp004643 | C41H70O14 | Majoroside R2 | 1.06 | 2.24 | up |
| Lmzp004896 | C36H62O10 | (24S)-Pseudo-ginsenoside RT4 | 1.08 | 2.19 | up |
| Lmzp005197 | C42H70O12 | Ginsenoside Rg5 | 1.07 | 0.12 | down |
| Lmzp005427 | C48H82O18 | Ginsenoside Rd | 1.07 | 0.11 | down |
| Lmzp006292 | C42H66O14 | Chikusetsusaponin IVa | 1.08 | 8680.70 | up |
| mws1553 | C42H72O14 | Ginsenoside Rg1 | 1.06 | 0.29 | down |
| mws1592 | C48H76O19 | Ginsenoside Ro | 1.07 | 2.96 | up |
| mws4022 | C42H72O13 | 20(S)-Ginsenoside Rg3 | 1.08 | 0.02 | down |
| mws4027 | C36H62O10 | Pseudoginsenoside RT5 | 1.08 | 2.26 | up |
| mws4031 | C42H70O12 | Ginsenoside Rk1 | 1.08 | 0.03 | down |
| mws4035 | C42H72O13 | Ginsenoside F2 | 1.08 | 0.04 | down |
| mws4036 | C36H62O9 | 20(S)-Ginsenoside Rh1 | 1.07 | 0.09 | down |
| pmn001506 | C36H58O8 | Oleanolic acid 2-O-β-D-glucopyranoside | 1.05 | 3.43 | up |

Supplementary Table 9: Accumulation of triterpenoid saponins in ZC and ZE

| Index | Formula | Compounds | VIP | Fold_Change | Type |
| --- | --- | --- | --- | --- | --- |
| Lmzn003299 | C54H92O23 | Ginsenoside Rb1 | 1.04 | 15.79 | up |
| Lmzn003820 | C44H74O15 | Yesanchinosides D | 1.04 | 0.22 | down |
| Lmzn003940 | C48H82O19 | Notoginsenoside G | 1.04 | 0.22 | down |
| Lmzn004178 | C48H82O19 | Majoroside F1 | 1.05 | 0.15 | down |
| Lmzn004497 | C58H98O26 | Notoginsenoside Fc | 1.00 | 0.42 | down |
| Lmzn004691 | C48H76O19 | Chikusetsusaponin V | 1.04 | 3.64 | up |
| Lmzn004931 | C42H66O14 | Zingibroside R1 | 1.05 | 92.99 | up |
| Lmzn005163 | C50H84O19 | 6″-Acetyl-ginsenoside Rd | 1.05 | 0.10 | down |
| Lmzp004417 | C36H62O9 | Ginsenoside Rf1 | 1.05 | 0.03 | down |
| Lmzp004450 | C42H72O15 | Majoroside R1 | 1.00 | 2.15 | up |
| Lmzp005197 | C42H70O12 | Ginsenoside Rg5 | 1.05 | 0.08 | down |
| Lmzp005427 | C48H82O18 | Ginsenoside Rd | 1.04 | 0.07 | down |
| Lmzp006292 | C42H66O14 | Chikusetsusaponin IVa | 1.05 | 13512.59 | up |
| mws1553 | C42H72O14 | Ginsenoside Rg1 | 1.05 | 0.07 | down |
| mws1592 | C48H76O19 | Ginsenoside Ro | 1.04 | 3.86 | up |
| mws1665 | C42H72O14 | Ginsenoside Rf | 1.04 | 0.33 | down |
| mws1666 | C48H82O18 | Ginsenoside Re | 1.03 | 0.47 | down |
| mws1721 | C47H80O18 | Notoginsenoside R1 | 1.02 | 0.22 | down |
| mws4022 | C42H72O13 | 20(S)-Ginsenoside Rg3 | 1.05 | 0.00 | down |
| mws4023 | C42H72O13 | Ginsenosides Rg2 | 1.04 | 0.18 | down |
| mws4031 | C42H70O12 | Ginsenoside Rk1 | 1.05 | 0.00 | down |
| mws4035 | C42H72O13 | Ginsenoside F2 | 1.05 | 0.04 | down |
| mws4036 | C36H62O9 | 20(S)-Ginsenoside Rh1 | 1.05 | 0.00 | down |
| pmn001505 | C41H64O13 | Oleanolic acid-3-O-β-D-pyran xylose(1→3)-β-D-pyran glucuronide | 1.05 | 77196.67 | up |
| pmn001506 | C36H58O8 | Oleanolic acid 2-O-β-D-glucopyranoside | 1.04 | 8.13 | up |

Supplementary Table 10: Accumulation of triterpenoid saponins in ZD and ZE

| Index | Formula | Compounds | VIP | Fold_Change | Type |
| --- | --- | --- | --- | --- | --- |
| Lmzn003299 | C54H92O23 | Ginsenoside Rb1 | 1.10 | 2.45 | up |
| Lmzn003820 | C44H74O15 | Yesanchinosides D | 1.11 | 0.25 | down |
| Lmzn003940 | C48H80O19 | Notoginsenoside G | 1.08 | 0.33 | down |
| Lmzn004178 | C48H82O19 | Majoroside F1 | 1.11 | 0.42 | down |
| Lmzn004931 | C42H66O14 | Zingibroside R1 | 1.11 | 2.36 | up |
| Lmzp004450 | C42H72O15 | Majoroside R1 | 1.08 | 0.44 | down |
| mws1553 | C42H72O14 | Ginsenoside Rg1 | 1.11 | 0.25 | down |
| mws1665 | C42H72O14 | Ginsenoside Rf | 1.11 | 0.31 | down |
| mws1721 | C47H80O18 | Notoginsenoside R1 | 1.08 | 0.41 | down |
| mws4022 | C42H72O13 | 20(S)-Ginsenoside Rg3 | 1.11 | 0.10 | down |
| mws4023 | C42H72O13 | Ginsenosides Rg2 | 1.11 | 0.29 | down |
| mws4031 | C42H70O12 | Ginsenoside Rk1 | 1.12 | 0.00 | down |
| mws4036 | C36H62O9 | 20(S)-Ginsenoside Rh1 | 1.12 | 0.00 | down |
| pmn001505 | C41H64O13 | Oleanolic acid-3-O-β-D-pyran xylose(1→3)-β-D-pyran glucuronide | 1.12 | 77196.67 | up |
| pmn001506 | C36H58O8 | Oleanolic acid 2-O-β-D-glucopyranoside | 1.10 | 2.37 | up |

Supplementary Table 11: KEGG database annotation

| KEGG_map | KEGG_level_1 | Description |
| --- | --- | --- |
| ko00945 | Metabolism | Stilbenoid, diarylheptanoid and gingerol biosynthesis |
| ko00940 | Metabolism | Phenylpropanoid biosynthesis |
| ko01110 | Metabolism | Biosynthesis of secondary metabolites |
| ko00073 | Metabolism | Cutin, suberine and wax biosynthesis |
| ko04626 | Organismal Systems | Plant-pathogen interaction |
| ko04075 | Environmental Information Processing | Plant hormone signal transduction |
| ko01100 | Metabolism | Metabolic pathways |
| ko04016 | Environmental Information Processing | MAPK signaling pathway - plant |
| ko00941 | Metabolism | Flavonoid biosynthesis |
| ko00195 | Metabolism | Photosynthesis |
| ko00196 | Metabolism | Photosynthesis - antenna proteins |
| ko00590 | Metabolism | Arachidonic acid metabolism |
| ko00909 | Metabolism | Sesquiterpenoid and triterpenoid biosynthesis |
| ko01040 | Metabolism | Biosynthesis of unsaturated fatty acids |
| ko00906 | Metabolism | Carotenoid biosynthesis |
| ko00860 | Metabolism | Porphyrin and chlorophyll metabolism |
| ko02010 | Environmental Information Processing | ABC transporters |
| ko00130 | Metabolism | Ubiquinone and other terpenoid-quinone biosynthesis |
| ko00950 | Metabolism | Isoquinoline alkaloid biosynthesis |
| ko00591 | Metabolism | Linoleic acid metabolism |
| ko00592 | Metabolism | alpha-Linolenic acid metabolism |
| ko04712 | Organismal Systems | Circadian rhythm - plant |
| ko00902 | Metabolism | Monoterpenoid biosynthesis |
| ko00062 | Metabolism | Fatty acid elongation |
| ko00460 | Metabolism | Cyanoamino acid metabolism |
| ko00040 | Metabolism | Pentose and glucuronate interconversions |
| ko00260 | Metabolism | Glycine, serine and threonine metabolism |
| ko00052 | Metabolism | Galactose metabolism |
| ko01212 | Metabolism | Fatty acid metabolism |
| ko00908 | Metabolism | Zeatin biosynthesis |
| ko00500 | Metabolism | Starch and sucrose metabolism |
| ko00514 | Metabolism | Other types of O-glycan biosynthesis |
| ko00402 | Metabolism | Benzoxazinoid biosynthesis |
| ko00430 | Metabolism | Taurine and hypotaurine metabolism |
| ko00604 | Metabolism | Glycosphingolipid biosynthesis - ganglio series |
| ko00900 | Metabolism | Terpenoid backbone biosynthesis |
| ko00942 | Metabolism | Anthocyanin biosynthesis |
| ko04146 | Cellular Processes | Peroxisome |
| ko00730 | Metabolism | Thiamine metabolism |
| ko00960 | Metabolism | Tropane, piperidine and pyridine alkaloid biosynthesis |
| ko00904 | Metabolism | Diterpenoid biosynthesis |
| ko00511 | Metabolism | Other glycan degradation |
| ko00100 | Metabolism | Steroid biosynthesis |
| ko00270 | Metabolism | Cysteine and methionine metabolism |
| ko00051 | Metabolism | Fructose and mannose metabolism |
| ko00710 | Metabolism | Carbon fixation in photosynthetic organisms |
| ko00261 | Metabolism | Monobactam biosynthesis |
| ko00531 | Metabolism | Glycosaminoglycan degradation |
| ko00790 | Metabolism | Folate biosynthesis |
| ko00660 | Metabolism | C5-Branched dibasic acid metabolism |
| ko00360 | Metabolism | Phenylalanine metabolism |
| ko00600 | Metabolism | Sphingolipid metabolism |
| ko00944 | Metabolism | Flavone and flavonol biosynthesis |
| ko00966 | Metabolism | Glucosinolate biosynthesis |
| ko00071 | Metabolism | Fatty acid degradation |
| ko00330 | Metabolism | Arginine and proline metabolism |
| ko00053 | Metabolism | Ascorbate and aldarate metabolism |
| ko00300 | Metabolism | Lysine biosynthesis |
| ko00311 | Metabolism | Penicillin and cephalosporin biosynthesis |
| ko03020 | Genetic Information Processing | RNA polymerase |
| ko00520 | Metabolism | Amino sugar and nucleotide sugar metabolism |
| ko00350 | Metabolism | Tyrosine metabolism |
| ko00565 | Metabolism | Ether lipid metabolism |
| ko00562 | Metabolism | Inositol phosphate metabolism |
| ko00290 | Metabolism | Valine, leucine and isoleucine biosynthesis |
| ko00561 | Metabolism | Glycerolipid metabolism |
| ko00010 | Metabolism | Glycolysis / Gluconeogenesis |
| ko00903 | Metabolism | Limonene and pinene degradation |
| ko00515 | Metabolism | Mannose type O-glycan biosynthesis |
| ko04130 | Genetic Information Processing | SNARE interactions in vesicular transport |
| ko00901 | Metabolism | Indole alkaloid biosynthesis |
| ko00480 | Metabolism | Glutathione metabolism |
| ko00061 | Metabolism | Fatty acid biosynthesis |
| ko00750 | Metabolism | Vitamin B6 metabolism |
| ko00400 | Metabolism | Phenylalanine, tyrosine and tryptophan biosynthesis |
| ko00905 | Metabolism | Brassinosteroid biosynthesis |
| ko01230 | Metabolism | Biosynthesis of amino acids |
| ko04122 | Genetic Information Processing | Sulfur relay system |
| ko00740 | Metabolism | Riboflavin metabolism |
| ko04070 | Environmental Information Processing | Phosphatidylinositol signaling system |
| ko00910 | Metabolism | Nitrogen metabolism |
| ko00943 | Metabolism | Isoflavonoid biosynthesis |
| ko00785 | Metabolism | Lipoic acid metabolism |
| ko00340 | Metabolism | Histidine metabolism |
| ko00410 | Metabolism | beta-Alanine metabolism |
| ko00280 | Metabolism | Valine, leucine and isoleucine degradation |
| ko00450 | Metabolism | Selenocompound metabolism |
| ko01502 | Human Diseases | Vancomycin resistance |
| ko00254 | Metabolism | Aflatoxin biosynthesis |
| ko00232 | Metabolism | Caffeine metabolism |
| ko04933 | Human Diseases | AGE-RAGE signaling pathway in diabetic complications |
| ko00230 | Metabolism | Purine metabolism |
| ko00920 | Metabolism | Sulfur metabolism |
| ko04120 | Genetic Information Processing | Ubiquitin mediated proteolysis |
| ko00030 | Metabolism | Pentose phosphate pathway |
| ko03018 | Genetic Information Processing | RNA degradation |
| ko03410 | Genetic Information Processing | Base excision repair |
| ko00240 | Metabolism | Pyrimidine metabolism |
| ko00380 | Metabolism | Tryptophan metabolism |
| ko00650 | Metabolism | Butanoate metabolism |
| ko00998 | Metabolism | Biosynthesis of secondary metabolites - other antibiotics |
| ko04136 | Cellular Processes | Autophagy - other |
| ko00440 | Metabolism | Phosphonate and phosphinate metabolism |
| ko01210 | Metabolism | 2-Oxocarboxylic acid metabolism |
| ko00072 | Metabolism | Synthesis and degradation of ketone bodies |
| ko00760 | Metabolism | Nicotinate and nicotinamide metabolism |
| ko00564 | Metabolism | Glycerophospholipid metabolism |
| ko00220 | Metabolism | Arginine biosynthesis |
| ko03450 | Genetic Information Processing | Non-homologous end-joining |
| ko03060 | Genetic Information Processing | Protein export |
| ko03015 | Genetic Information Processing | mRNA surveillance pathway |
| ko00250 | Metabolism | Alanine, aspartate and glutamate metabolism |
| ko00601 | Metabolism | Glycosphingolipid biosynthesis - lacto and neolacto series |
| ko00620 | Metabolism | Pyruvate metabolism |
| ko00310 | Metabolism | Lysine degradation |
| ko00780 | Metabolism | Biotin metabolism |
| ko00770 | Metabolism | Pantothenate and CoA biosynthesis |
| ko01200 | Metabolism | Carbon metabolism |
| ko03022 | Genetic Information Processing | Basal transcription factors |
| ko00630 | Metabolism | Glyoxylate and dicarboxylate metabolism |
| ko03008 | Genetic Information Processing | Ribosome biogenesis in eukaryotes |
| ko00965 | Metabolism | Betalain biosynthesis |
| ko00670 | Metabolism | One carbon pool by folate |
| ko00563 | Metabolism | Glycosylphosphatidylinositol (GPI)-anchor biosynthesis |
| ko03440 | Genetic Information Processing | Homologous recombination |
| ko00513 | Metabolism | Various types of N-glycan biosynthesis |
| ko00603 | Metabolism | Glycosphingolipid biosynthesis - globo and isoglobo series |
| ko00640 | Metabolism | Propanoate metabolism |
| ko03040 | Genetic Information Processing | Spliceosome |
| ko04144 | Cellular Processes | Endocytosis |
| ko03050 | Genetic Information Processing | Proteasome |
| ko00970 | Genetic Information Processing | Aminoacyl-tRNA biosynthesis |
| ko03420 | Genetic Information Processing | Nucleotide excision repair |
| ko00190 | Metabolism | Oxidative phosphorylation |
| ko03030 | Genetic Information Processing | DNA replication |
| ko04141 | Genetic Information Processing | Protein processing in endoplasmic reticulum |
| ko03430 | Genetic Information Processing | Mismatch repair |
| ko04145 | Cellular Processes | Phagosome |
| ko00510 | Metabolism | N-Glycan biosynthesis |
| ko00020 | Metabolism | Citrate cycle (TCA cycle) |
| ko03013 | Genetic Information Processing | RNA transport |
| ko03010 | Genetic Information Processing | Ribosome |
